# Identification and preliminary validation of biomarkers associated with mitochondrial and programmed cell death in pre-eclampsia

**DOI:** 10.3389/fimmu.2024.1453633

**Published:** 2025-01-23

**Authors:** Rong Lin, XiaoYing Weng, Liang Lin, XuYang Hu, ZhiYan Liu, Jing Zheng, FenFang Shen, Rui Li

**Affiliations:** ^1^ Medical Centre of Maternity and Child Health, Shengli Clinical Medical College of Fujian Medical University, Fuzhou, Fujian, China; ^2^ Fuzhou University Affiliated Provincial Hospital, Fuzhou, Fujian, China

**Keywords:** mitochondrial, programmed cell death, pre-eclampsia, bioinformatics, database

## Abstract

**Background:**

The involvement of mitochondrial and programmed cell death (mtPCD)–related genes in the pathogenesis of pre-eclampsia (PE) remains inadequately characterized.

**Methods:**

This study explores the role of mtPCD genes in PE through bioinformatics and experimental approaches. Differentially expressed mtPCD genes were identified as potential biomarkers from the GSE10588 and GSE98224 datasets and subsequently validated. Hub genes were determined using support vector machine, least absolute shrinkage and selection operator, and Boruta based on consistent expression profiles. Their performance was assessed through nomogram and artificial neural network models. Biomarkers were subjected to localization, functional annotation, regulatory network analysis, and drug prediction. Clinical validation was conducted *via* real-time quantitative polymerase chain reaction (RT-qPCR), immunofluorescence, and Western blot.

**Results:**

Four genes [solute carrier family 25 member 5 (*SLC25A5*), acyl-CoA synthetase family member 2 (*ACSF2*), mitochondrial fission factor (*MFF*), and phorbol-12-myristate-13-acetate–induced protein 1 (*PMAIP1*)] were identified as biomarkers distinguishing PE from normal controls. Functional analysis indicated their involvement in various biological pathways. Immune analysis revealed associations between biomarkers and immune cell activity. A regulatory network was informed by biomarker expression and database predictions, in which *KCNQ1OT1* modulates *ACSF2* expression *via* hsa-miR-200b-3p. Drug predictions, including clodronic acid, were also proposed. Immunofluorescence, RT-qPCR, and Western blot confirmed reduced expression of *SLC25A5, MFF*, and *PMAIP1* in PE, whereas *ACSF2* was significantly upregulated.

**Conclusion:**

These four mtPCD-related biomarkers may play a pivotal role in PE pathogenesis, offering new perspectives on the disease’s diagnostic and mechanistic pathways.

## 1 Introduction

Pre-eclampsia (PE) is a distinct, progressive multisystem disorder that typically arises after 20 weeks of gestation, characterized by hypertension and proteinuria (1). It can lead to complications such as fetal growth restriction, fetal distress, and preterm birth, with severe cases resulting in stillbirth and neonatal death (2). The global incidence of PE is approximately 5% to 8% (3, 4), causing an estimated 75,000 maternal deaths and 500,000 neonatal deaths annually, making it the second leading cause of maternal mortality. New-onset hypertension is a key diagnostic criterion for PE (5). The condition is classified into early-onset PE, which occurs before 34 weeks of gestation, and late-onset PE, which occurs at or after 34 weeks (6). Although extensive studies have linked PE pathogenesis to placental hypoxia and ischemia, oxidative stress, inflammatory responses, angiogenesis, functional imbalance, and immune dysregulation (7), the exact mechanisms remain incompletely understood. Current management focuses on controlling hypertension and monitoring maternal and fetal health, with the only effective treatment being the termination of pregnancy (8). However, premature termination increases the risks associated with preterm birth, jeopardizing both maternal and fetal health (9). Although soluble fms-like tyrosine kinase 1 (sFlt-1) and placental growth factor (*PlGF*) have been explored as screening markers for PE (10, 11), their predictive value remains suboptimal. The sFlt-1/*PlGF* ratio rises significantly both prior to and during the clinical onset of PE. While its negative predictive value is as high as 99%, its positive predictive value is limited to just 36.7%, indicating its insufficient efficacy in predicting PE onset (12, 13). Furthermore, variability in testing methods across laboratories compromises the reliability and accuracy of the sFlt-1/*PlGF* ratio (14). Differences in detection protocols can introduce measurement biases, affecting the interpretation of the ratio. Moreover, by the time an elevated sFlt-1/*PlGF* ratio is detected, most patients have already developed clinical symptoms of PE, limiting its utility for early prediction and intervention. Given these challenges, there is an urgent need to identify novel biomarkers with high specificity and sensitivity that could not only predict PE but also serve as potential therapeutic targets for early intervention.

Two main forms of cell death—accidental cell death and programmed cell death (PCD)—are recognized. PCD is the primary mode of cell death, a vital physiological process that is tightly regulated by multiple mechanisms and plays a pivotal role in eliminating damaged or unnecessary cells to maintain tissue homeostasis (15). The term “programmed cell death” was coined by Richard Lockshin and Carroll M. Williams in the 1960s (16) and primarily refers to apoptosis, necroptosis, and pyroptosis. Other forms include ferroptosis, cuproptosis, autophagy, endocytosis, disulfidptosis, lysosomal cell death, and cytotoxicity (17). PCD is implicated in numerous diseases, including cancer, cardiovascular disorders, inflammation, and neurodegenerative diseases (18–20). Recent research on the pathogenesis of PE has increasingly focused on trophoblast programmed death. Trophoblast cell necrosis has been shown to reduce cell viability; increase mortality; impair migration, invasion, and tube formation; and promote cell fusion. These alterations disrupt spiral artery remodeling, leading to placental dysfunction and the progression of PE (21). Several studies suggest that trophoblast PCD contributes to placental insufficiency, which, in turn, causes PE (22–25). With advancing research on PCD mechanisms, various drugs targeting these pathways have been clinically developed, demonstrating significant potential in cancer treatment. For instance, Venetoclax (a *BCL-2*–specific inhibitor) and Navitoclax (inhibitors of *BCL-2, BCL-xL*, and *BCL-W*) have shown promise in the treatment of leukemia and lymphoma (26, 27). However, current research on PE prediction and treatment has largely overlooked the detailed exploration of trophoblast PCD. Despite significant progress in understanding related mechanisms in cancer therapy, there remains considerable opportunity for investigation in the context of PE. Thus, a deeper study of PCD may offer new insights and therapeutic strategies for the prediction and treatment of PE.

Since the first description of PCD 60 years ago, numerous studies have confirmed the involvement of mitochondria in PCD, identifying them as key regulators in triggering this process. Mitochondria are ubiquitous, double-membrane-bound organelles that regulate cellular energy production, support cell activities, modulate cellular metabolic pathways, which even mediate cell fate decisions. They can participate directly or indirectly in PCD through various mechanisms and pathways, influencing the onset and progression of numerous human diseases. Mitochondria-related PCD is extensively involved in the pathological progression of diseases across different organ systems (28). An observational study first reported in 1989 found a high prevalence of PE in families with mitochondrial dysfunction (29). Since then, mitochondrial dysfunction in the placenta has been demonstrated in both pregnant women with PE and animal models of the condition (30). Over the past 30 years, compelling evidence has shown that abnormal mitochondrial function is a major contributor to placental dysfunction, and it is well established that PE arises from placental dysfunction, although the exact cause of PE remains unclear. Mitochondrial dysregulation caused by placental hypoxia is typically characterized by increased mitochondrial reactive oxygen species (ROS), mitochondrial fission, mitophagy, and apoptosis, along with reduced release of bioactive factors from the placenta. These alterations lead to placental and vascular endothelial dysfunction, ultimately driving the development of PE (31). Furthermore, oxidative stress is a critical factor in this process. As key organelles are responsible for intracellular energy supply, mitochondria are highly vulnerable to functional damage, which can disrupt energy metabolism (32). In PE, mitochondrial oxidative phosphorylation may be impaired, leading to reduced ATP production (33). Additionally, maintaining metabolic turnover balance is essential for immune cell function, and mitochondrial dysfunction can disturb this balance, compromising immune cell activity (34). Mitochondrial dysregulation and dysfunction induced by placental hypoxia and oxidative stress play a pivotal role in the pathogenesis of PE, from impairing placental and vascular endothelial cell function to disrupting immune cell metabolism. These changes highlight the critical need for in-depth research into mitochondria-related mechanisms to fully understand the pathogenesis of PE. Further exploration of the role of mitochondria and PCD in PE is essential to better understand their interaction in disease development, providing vital insights for the development of new therapeutic strategies and diagnostic tools.

In conclusion, PCD and mitochondrial dysfunction play central roles in the pathogenesis of PE. To further investigate the intersection of these two factors, this study explored potential molecular mechanisms associated with PE biomarkers using the Gene Expression Omnibus (GEO) database (GSE10588 and GSE98224). The study assessed the predictive efficacy of these biomarkers for PE and proposed drug predictions, offering new targets and strategies for the diagnosis and treatment of PE. Additionally, real-time quantitative polymerase chain reaction (RT-qPCR) validation was conducted to confirm the findings, reinforcing the significance of the identified biomarkers and their potential roles in PE.

## 2 Materials and methods

### 2.1 Data extraction

In this study, the GSE10588 dataset (35) and GSE98224 dataset (36) were retrieved from the GEO database (https://www.ncbi.nlm.nih.gov/geo/). The GSE10588 dataset included 17 placental tissue samples from patients with PE and 26 placental tissue samples from normal individuals, serving as the training set. For validation, the GSE98224 dataset consisted of 30 placental tissue samples from patients with PE and 18 placental tissue samples from normal individuals, acting as the validation set. A total of 1,136 mitochondrial-related genes (MRGs) were obtained from the MitoCarta 3.0 database, and 1,548 programmed cell death–related genes (PCDs) were collected from the literature.

### 2.2 Identification of DE-mtPCDs

Differentially expressed genes (DEGs) in the GSE10588 dataset were identified using the limma package (37) (v 3.56.2). The criteria for defining DEGs were an adjusted p-value <0.05 and |log2 fold change (log2FC)| >0.5. The DEGs were then intersected with MRGs to obtain differentially expressed MRGs (DE-MRGs). Following this, the DEGs were intersected with PCDs to identify differentially expressed PCDs (DE-PCDs). Finally, the intersection of DE-MRGs and DE-PCDs was used to identify differentially expressed mitochondrial PCD–related genes (DE-mtPCD).

### 2.3 Functional enrichment analysis of DE-mtPCDs

To explore the potential roles of the DE-mtPCD, functional enrichment analysis was conducted using the ClusterProfiler package (38) (v 4.8.3), which included Gene Ontology (GO) and Kyoto Encyclopedia of Genes and Genomes (KEGG) pathway enrichment analyses. The DE-mtPCDs were subjected to both GO and KEGG analyses, with a filtering criterion of p.adjust <0.05.

### 2.4 Protein–protein interaction analysis and visualization of DE-mtPCDs

Protein–protein interaction (PPI) analysis was performed on the DE-mtPCD by inputting the genes into the STRING database (https://string-db.org/), using an interaction score threshold of 0.4 to exclude low-confidence interactions. The resulting network was visualized using Cytoscape software (39) (v 3.9.1).

### 2.5 Identification of hub genes among DE-mtPCDs

To further identify potential hub genes within the DE-mtPCD, three machine learning algorithms were employed: support vector machine (SVM), least absolute shrinkage and selection operator (LASSO), and Boruta. Feature genes were screened using the mlbench package, glmnet package (40) (v 4.1-2), and Boruta package (v 8.0.0), respectively. The intersection of the feature genes identified by all three algorithms was defined as the hub genes.

### 2.6 Validation and selection of biomarker genes

To validate the ability of the identified hub genes to distinguish between PE and normal samples and to identify suitable biomarkers, a Wilcoxon test was performed to confirm their differential expression in both the GSE10588 and GSE98224 datasets. Genes that exhibited significant and consistent expression patterns in both datasets were considered potential biomarkers.

### 2.7 Nomogram model construction and performance evaluation

To elucidate the relationship between each biomarker and PE onset, the rms package (v 6.2-0) (41) was used to construct a Nomogram model based on multivariable logistic regression. Additionally, the diagnostic potential of sFlt-1 and *PlGF* biomarkers, previously confirmed in the literature, was explored by constructing nomogram models for the *PlGF* (PGF) and sFlt-1 (FLT1) genes in the GSE10588 and GSE98224 datasets. Calibration curves were generated to assess the reliability and accuracy of the model predictions. To further verify the model performance, receiver operating characteristic (ROC) curves were plotted using the pROC package (v 1.18.4) (42) for both datasets.

### 2.8 ANN diagnostic model construction and performance evaluation

To assess whether the biomarkers can distinguish between PE and normal samples in the GSE10588 dataset, an artificial neural network (ANN) diagnostic model based on logistic regression was developed using the neuralnet package (v 1.44.2) (43). ROC curves were subsequently generated on the basis of the predicted results from the GSE10588 dataset to assess diagnostic performance. Additionally, biomarker expression levels from the GSE98224 dataset were input into the trained ANN model, and ROC curves were plotted to evaluate the model’s accuracy.

### 2.9 Biomarker genomic localization analysis

Gene chromosomal localization is essential for understanding gene function, studying genetic diseases, and advancing gene therapy and genomics. The OmicCircos package was used to analyze and visualize the genomic localization of biomarkers on chromosomes.

### 2.10 Biomarker subcellular localization prediction and analysis

To further investigate the subcellular localization of biomarkers and their relevance to PE pathogenesis, the mRNALocater online tool (http://bio-bigdata.cn/mRNALocater/) was employed to predict the subcellular localization of biomarkers in the GSE10588 dataset. Subcellular localization scores were also calculated for the biomarkers.

### 2.11 GSVA

To explore the impact of differential biomarker expression on KEGG pathways, the GSE10588 dataset was divided into high- and low-expression groups on the basis of the median expression levels of biomarkers. Gene set variation analysis (GSVA) scores for KEGG pathways were then calculated between the two expression groups using the c2.cp.kegg.v2023.1.Hs.symbols.gmt as a background gene set. Differential signaling pathways were identified on the basis of the criteria |t| > 2 and p < 0.05 using the limma package (v 3.56.2) (37).

### 2.12 Immune microenvironment analysis

To analyze the immune microenvironment of PE and normal samples, the Single Sample Gene Set Enrichment Analysis (ssGSEA) algorithm from the GSVA package (v 1.42.0) (44) was used to calculate scores for 28 immune cell compositions based on gene expression profiles from the GSE10588 dataset. Differential analysis using the Wilcoxon test was then performed to identify immune cell types with differential expression. Additionally, Spearman correlation analysis was conducted to explore the relationship between biomarkers and the identified immune cell types.

### 2.13 Construction of regulatory network

To explore the regulatory mechanisms of the aforementioned biomarkers, miRDB (http://mirdb.org/) and miRTarBase (https://awi.cuhk.edu.cn/~miRTarBase/miRTarBase_2025/php/index.php) databases were employed to predict miRNAs that regulate these biomarkers. In miRDB, miRNAs with a Target score ≥80 were selected, whereas, for miRTarBase, only miRNAs with reported experimental evidence were considered. The overlap between these two sets of miRNAs was determined to identify shared regulatory miRNAs. Subsequently, StarBase (http://starbase.sysu.edu.cn/) and miRNet (https://www.mirnet.ca/) databases were utilized to predict the long non-coding RNAs (lncRNAs) interacting with the shared miRNAs. The intersection of predicted lncRNAs from both databases was used to obtain common lncRNAs. A regulatory network consisting of lncRNAs, miRNAs, and mRNAs was then constructed. Cytoscape software (v 3.9.1) (39) was employed to visualize and analyze the resulting complex biological network.

### 2.14 Drug prediction

For the identification of potential drugs targeting biomarkers for PE treatment, drug prediction was carried out by integrating results from DrugBank (https://www.drugbank.ca/) and the Drug-Gene Interaction (DGI) database (http://www.dgidb.org/), resulting in a comprehensive list of candidate drugs. The relationships between drugs and biomarkers were represented in a drug-biomarker network, which was also visualized using Cytoscape (v 3.9.1) (39).

### 2.15 Validation of the biological indicators of the screening

#### 2.15.1 Sample collection

##### 2.15.1.1 Study objects

This research was conducted at the Obstetrics and Gynecology Department of Fujian Provincial Hospital (Jinshan Branch). Ten pregnant women scheduled for delivery were enrolled and classified into two groups: the experimental group, comprising five individuals diagnosed with PE, and the control group, consisting of five women who underwent cesarean deliveries for reasons unrelated to medical complications, such as social considerations or non-vertex fetal presentations (e.g., transverse or breech positions).

##### 2.15.1.2 Specimen collection

Approximately 40 g of placental tissue were collected, with large vessels and connective tissue removed. The tissue was thoroughly washed with 0.9% saline until the wash solution became nearly colorless, then finely minced into 1- to 3-mm pieces, preserved in a sterile isotonic solution, and subsequently frozen at −80°C.

The study adhered to the Declaration of Helsinki and received approval from the Ethics Committee of Fujian Provincial Hospital (protocol code K2023 - 02 - 016, approval date: 22 February 2023). Informed consent was obtained from all participants.

#### 2.15.2 Real-time quantitative polymerase chain reaction

Total RNA was extracted from the placental tissue samples of five control and five patients with PE using TRIzol solution (Ambion, Austin, USA), following the manufacturer’s instructions. The RNA concentration was determined using NanoDrop, and complementary DNA (cDNA) was synthesized with the SweScript First Strand cDNA Synthesis Kit (Servicebio, Wuhan, China). The reverse transcription conditions were as follows: primer-template binding at 25°C for 5 min, cDNA synthesis at 50°C for 15 min, and denaturation of the first strand from mRNA at 58°C, followed by storage at 4°C. Primers for RT-qPCR were designed and synthesized (**Table 1**). Quantitative analysis was performed using the CFX96 real-time quantitative fluorescence PCR machine (BIO-RAD, California, USA). The reaction conditions included an initial denaturation at 95°C for 1 min, followed by 40 cycles of denaturation at 95°C for 20 s, annealing at 55°C for 20 s, and extension at 72°C for 30 s. Amplification and melting curves were generated, and the Cycle threshold (Ct) values were recorded. Glyceraldehyde-3-phosphate dehydrogenase (GAPDH) was used as an internal control, and gene expression levels were calculated using the 2^−ΔΔCt^ method.

**Table 1:** Related primer sequences.

Primer	Sequences
*SLC25A5* F	AGACTGCGTGGTCCGTATTC
*SLC25A5* R	TGCCAGATTCCCTGCAAAGT
*ACSF2* F	CTGTGAACCCAGCCTACCAG
*ACSF2* R	GCTGGGCATTCTCCACTTCT
*MFF* F	AACCCCTGGCACTGAAAACA
*MFF* R	TGCCAACTGCTCGGATTTCT
*PMAIP1* F	CCAAGCCGGATTTGCGATTG
*PMAIP1* R	CTCCTAGAGACAGCCGCCTA
GAPDH F	CGAAGGTGGAGTCAACGGATTT
GAPDH R	ATGGGTGGAATCATATTGGAAC

#### 2.15.3 Immunofluorescence assay

Tissue samples were fixed in 4% paraformaldehyde and embedded in paraffin. Deparaffinized and rehydrated tissue sections were permeabilized with 0.1% Triton X-100 in Phosphate Buffer Saline (PBS) for 5 min and blocked with 10% goat serum for 1 hour. Antigen retrieval was performed by heating the sections in Ethylenediaminetetraacetic Acid (EDTA)-Tris buffer [50 mM Tris and 1 mM EDTA (pH 9.0)]. After washing, the sections were incubated with primary antibodies overnight at 4°C, followed by incubation with fluorescent secondary antibodies. The slides were mounted with a 4',6-diamidino-2-phenylindole (DAPI)-containing medium and observed under a laser confocal microscope (Olympus Corporation, Japan).

#### 2.15.4 Western blot assay

Proteins were extracted from tissues of each experimental group. These proteins were then separated using 10% sodium dodecyl sulfate–polyacrylamide gel electrophoresis and subsequently transferred onto a membrane. The membrane was blocked to prevent non-specific binding, followed by incubation with the corresponding primary antibodies against solute carrier family 25 member 5 (*SLC25A5*), acyl-CoA synthetase family member 2 (*ACSF2*), mitochondrial fission factor (*MFF*), and phorbol-12-myristate-13-acetate–induced protein 1 (*PMAIP1*) overnight. After incubation with the primary antibodies, the membrane was incubated with the appropriate secondary antibodies and then exposed to detect the protein bands.

### 2.16 Statistical analysis

Data were processed and analyzed using R software and GraphPad Prism version 9.0. Differences between groups were assessed using t-tests, the Wilcoxon rank-sum test, and one-way ANOVA, with p-values <0.05 considered statistically significant.

## 3 Results

### 3.1 Identification of DEGs and DE-mtPCDs in PE

**Figure 1:**
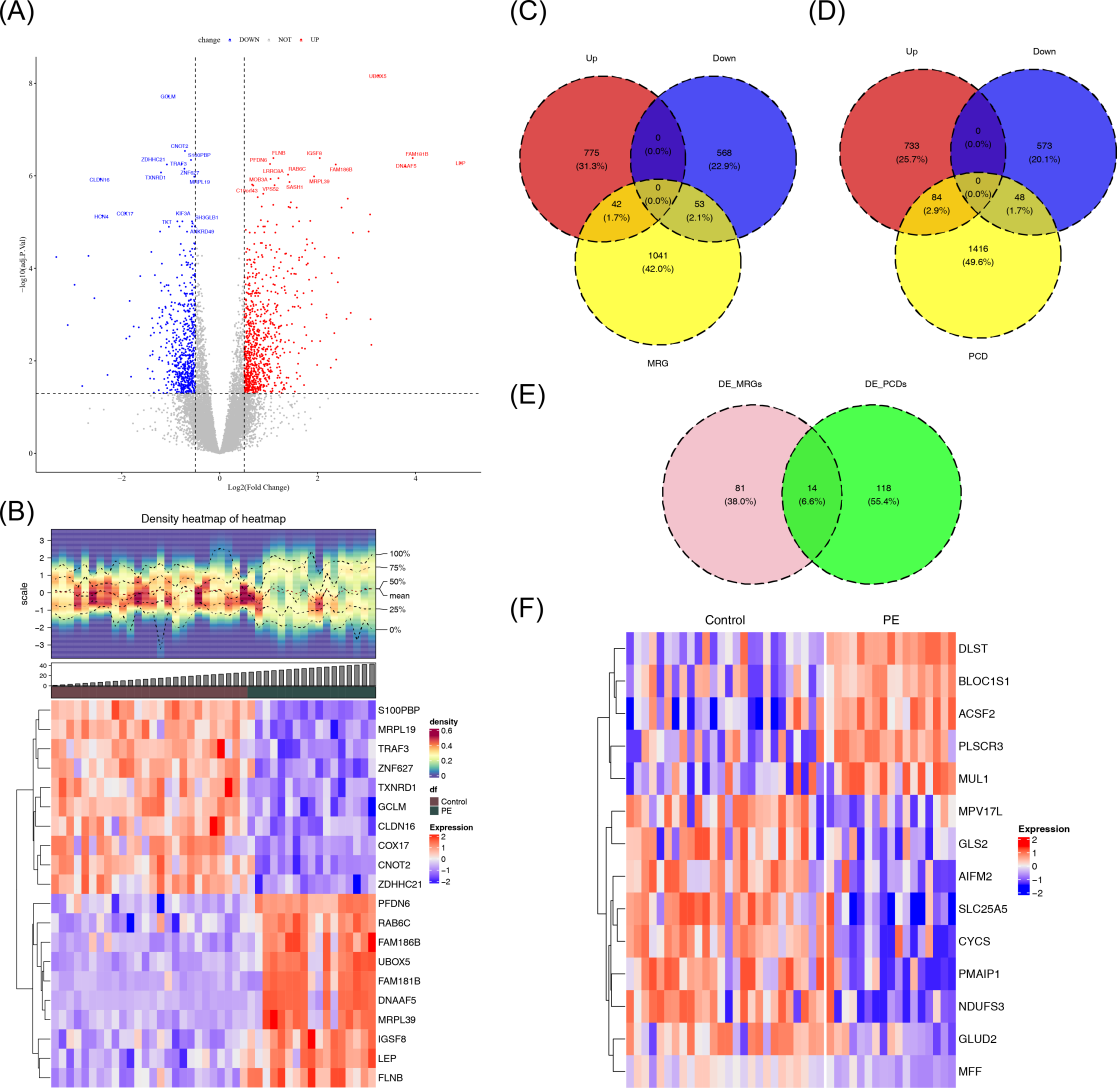
Identification of DE-mtPCDs in the GSE10588 dataset. **(A)** Volcano plot of the DEGs, with downregulated genes in blue, upregulated genes in red, and genes with insignificant differences in gray. **(B)** Heatmap of the DEGs, with the upper section displaying the expression density heatmap of differentially expressed genes across samples, showing lines for the five quantiles and mean values, and the lower section indicating high expression in red and low expression in blue. **(C)** Venn diagram of DE-MRGs. **(D)** Venn diagram of DE-PCDs. **(E)** Venn diagram of DE-mtPCDs. **(F)** Heatmap of DE-mtPCDs, with red indicating high expression and blue indicating low expression. DEGs, differentially expressed genes; DE-MRGs, differentially expressed mitochondrial–related genes; DE-PCDs, differentially expressed programmed cell death genes; DE-mtPCDs, differentially expressed mitochondrial and programmed cell death genes.

A total of 1,438 DEGs were identified in the GSE10588 dataset between the PE and normal groups, with 817 genes upregulated and 621 genes downregulated in the PE group (**Figures 1A, B**). A total of 95 DE-MRGs were identified by intersecting the DEGs with MRGs, of which 42 were upregulated and 53 were downregulated in the PE group. Additionally, 132 DE-PCDs were obtained by intersecting the DEGs with PCDs, with 84 upregulated and 48 downregulated in the PE group. By further intersecting the DE-MRGs with the DE-PCDs, 14 DE-mtPCDs were identified, comprising five upregulated and nine downregulated genes in the PE group (**Figures 1C–F**).

### 3.2 Functional and pathway enrichment of DE-mtPCDs in PE

**Figure 2:**
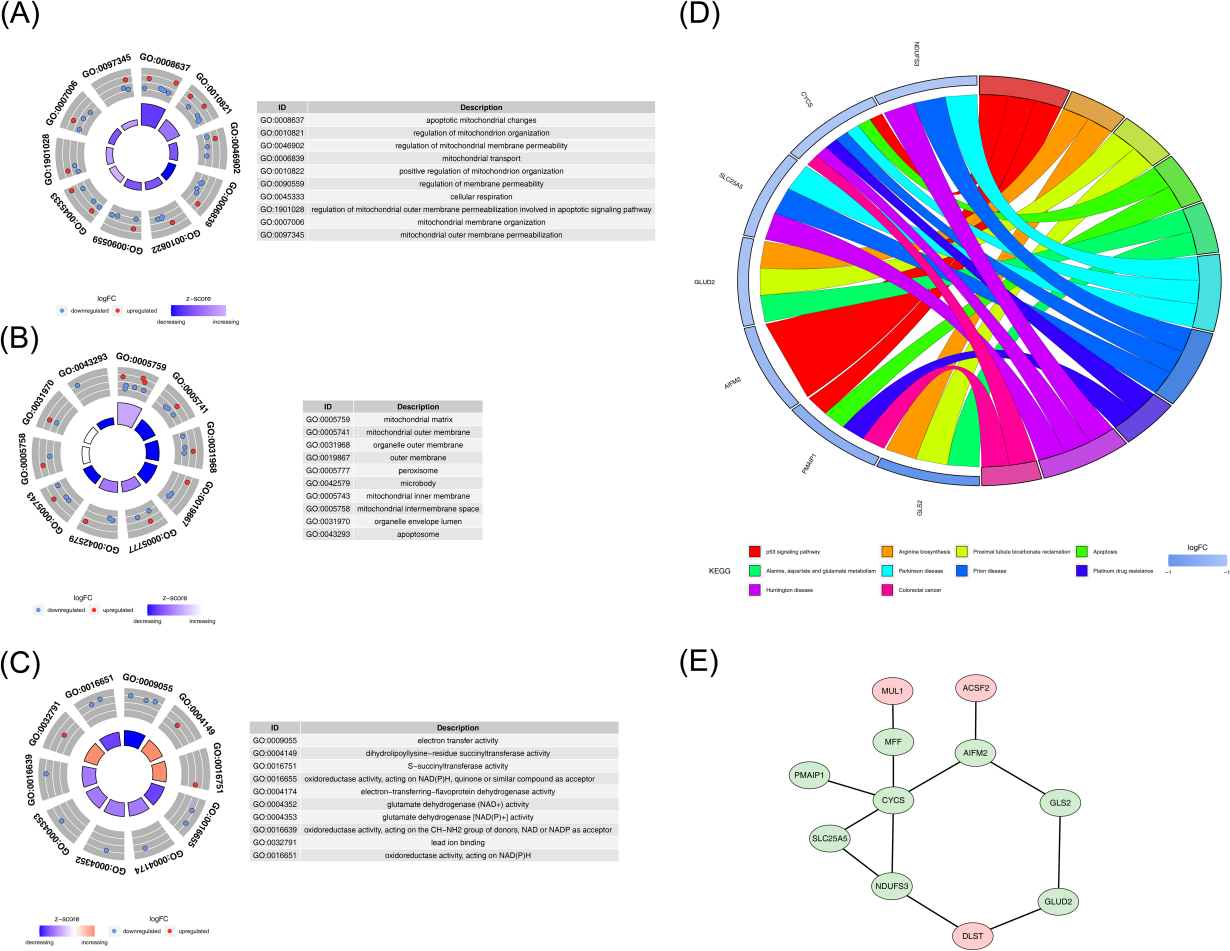
Functional enrichment analysis of DE-mtPCDs. **(A)** Enriched GO terms for DE-mtPCDs in Biological Process. **(B)** Cell Component. **(C)** Molecular Function. **(D)** KEGG enrichment results for DE-mtPCDs. **(E)** Protein–protein interaction network of DE-mtPCDs, with gene labels represented by nodes; red indicates upregulated genes, and green indicates downregulated genes; each line represents an interaction between genes. DE-mtPCDs, differentially expressed mitochondrial and programmed cell death genes; KEGG, Kyoto Encyclopedia of Genes and Genomes.

To explore the biological functions and pathways associated with the identified DE-mtPCDs, an enrichment analysis was performed. The analysis revealed 225 enriched GO terms, including 160 Biological Process (BP), 24 Cellular Component (CC), and 41 Molecular Function (MF) terms. The BP enrichment analysis indicated that the DE-mtPCDs were significantly enriched in mitochondrial functions and apoptosis-related processes, such as regulation of mitochondrial membrane permeability and apoptotic mitochondrial changes (**Figure 2A**). The CC analysis showed that the DE-mtPCDs were primarily localized in membrane structures of organelles, including peroxisomes and the mitochondrial inner membrane (**Figure 2B**). The MF analysis revealed that the DE-mtPCDs predominantly affected oxidative-reduction processes involved in electron transfer activity (**Figure 2C**). KEGG pathway analysis further indicated enrichment in 23 metabolic pathways, including several apoptosis-related pathways such as the p53 signaling pathway and apoptosis pathway, as well as disease-associated pathways like Huntington’s disease and prion diseases (**Figure 2D**). A PPI network constructed with the 14 DE-mtPCDs, consisting of 11 nodes and 12 edges, revealed multiple interactions among these proteins, including cytochrome c (*CYCS*) with *MFF*, *PMAIP1*, *SLC25A5*, Nicotinamide Adenine Dinucleotide (Reduced) Hydrogen (NADH) dehydrogenase (ubiquinone) Fe-S protein 3 (NDUFS3), and apoptosis-inducing factor mitochondria-associated 2 (AIFM2) (**Figure 2E**).

### 3.3 Identification of Hub genes in PE

**Figure 3:**
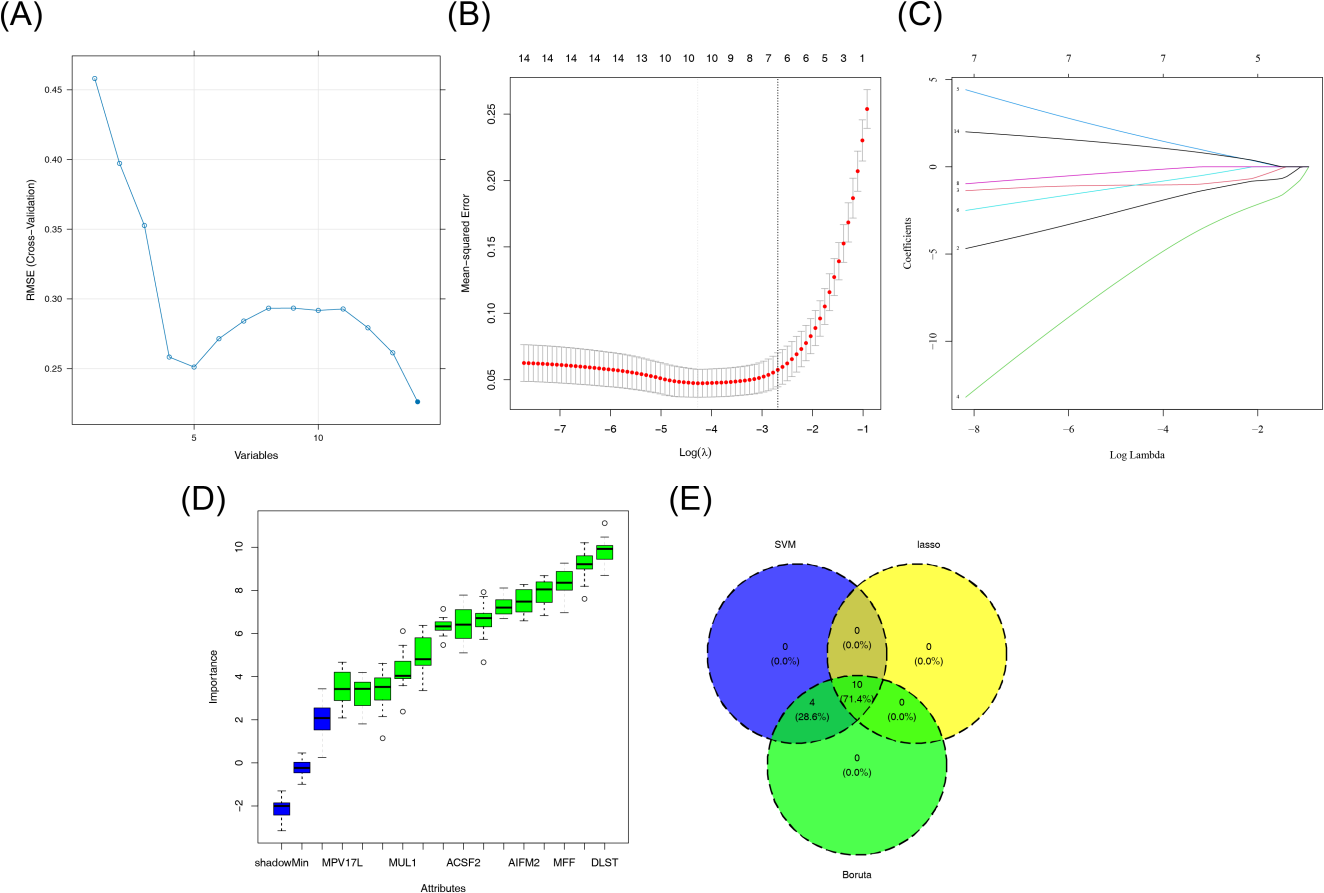
Machine learning screening results. **(A)** SVM classification results. **(B, C)** LASSO regression results. **(D)** Boruta algorithm results. **(E)** Venn diagram showing the crossover genes between LASSO, SVM, and Boruta. LASSO, least absolute shrinkage and selection operator; SVM, support vector machine.

Based on the 14 DE-mtPCDs, the SVM algorithm was used to screen 14 feature genes (**Figure 3A**). After 10-fold cross-validation, 10 feature genes were identified in the LASSO regression model, with dihydrolipoamide S-succinyltransferase (*DLST*), *CYCS, SLC25A5, NDUFS3, ACSF2, MPV17* mitochondrial inner membrane protein like (*MPV17L*), glutaminase 2 (*GLS2*), *MFF, PMAIP1*, and phospholipid scramblase 3 (*PLSCR3*) selected, achieving the lowest error rate at the optimal lambda.best parameter value of 0.01397921 (**Figures 3B, C**). Additionally, the Boruta algorithm identified 14 feature genes, which scored significantly higher than the maximum Z-score among shadow attributes (median, 2.077892) (**Figure 3D**). To establish a consensus set of feature genes, the intersection of the feature genes obtained from the three algorithms was taken, resulting in a final set of 10 hub genes. These hub genes included *DLST, CYCS, SLC25A5, NDUFS3, ACSF2, MPV17L, GLS2, MFF, PMAIP1*, and *PLSCR3* (**Figure 3E**; **Supplementary Table S1**).

### 3.4 Biomarker discovery and localization in PE

**Figure 4:**
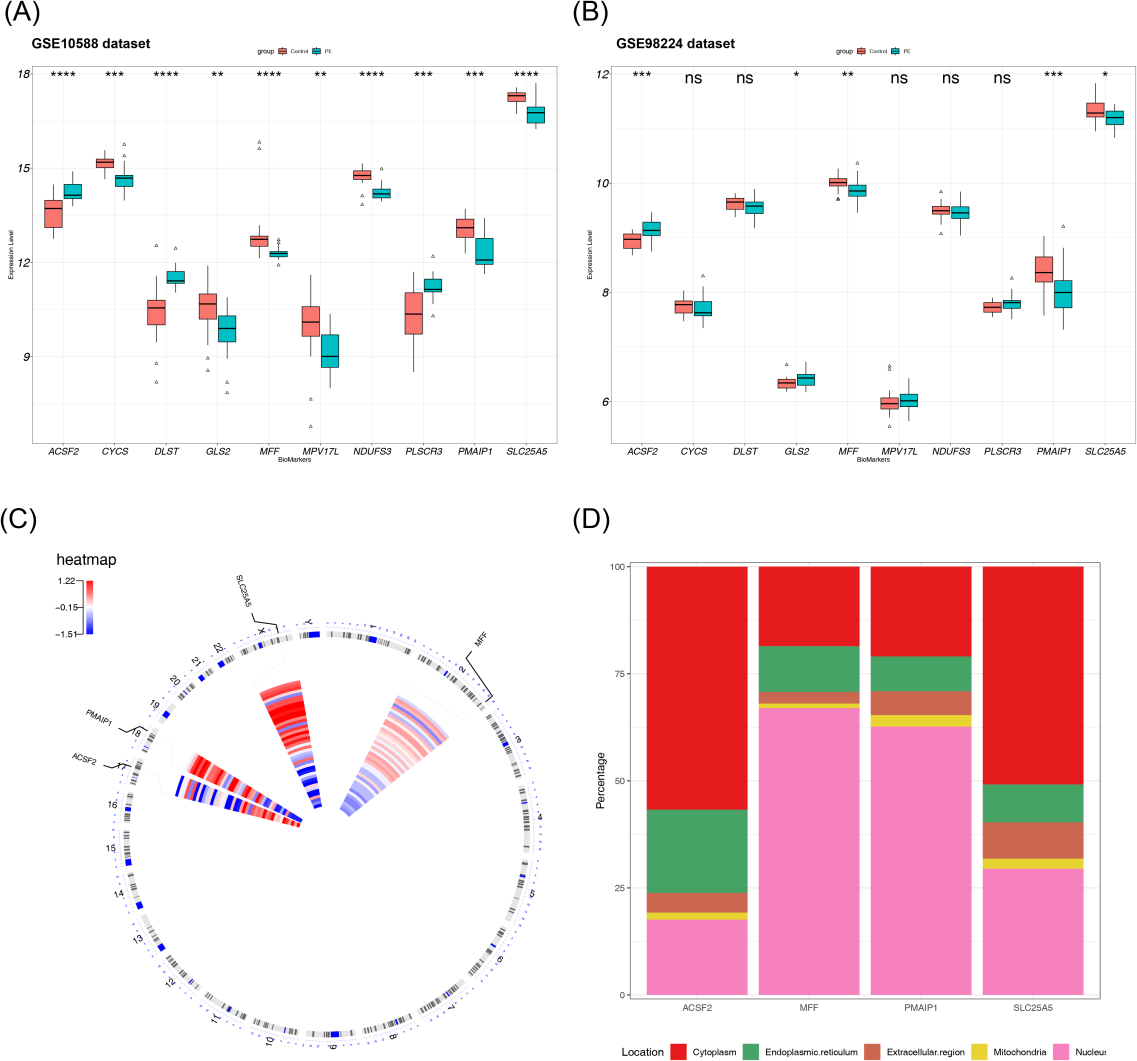
Biomarker discovery and localization. **(A)** Expression distribution of 10 candidate biomarkers in the GSE10588 dataset, with differences in expression verified using a rank sum test. **(B)** Expression distribution of 10 candidate biomarkers in the GSE98224 dataset. **(C)** Chromosomal localization of the biomarkers. **(D)** Subcellular structural localization of the biomarkers. NS, non-significant, p > 0.05; *p < 0.05; **p < 0.01; ***p < 0.001; ****p < 0.0001.

To validate the discriminatory ability of the candidate biomarkers in distinguishing PE samples from normal samples and to select suitable genes as biomarkers, the expression of the 10 hub genes was analyzed across two datasets. The final results revealed that 5 of the 10 hub genes showed differential expression between PE and control samples in both datasets. However, the expression trend of *GLS2* was inconsistent across the datasets, leading to its exclusion. Therefore, four genes—*SLC25A5, ACSF2, MFF*, and *PMAIP1*—were selected for further investigation due to their significant differential expression and consistent trends in both the GSE10588 and GSE98224 datasets. Among these, *SLC25A5*, *MFF*, and *PMAIP1* exhibited low expression in the PE group, whereas *ACSF2* displayed an opposite trend in the two datasets. Thus, these four genes were identified as final biomarkers for PE (**Figures 4A, B**). To investigate the chromosomal locations of the four biomarkers, the chromosomal positions for each biomarker were analyzed. In summary, the analysis revealed that the chromosomal locations of the four biomarkers were also analyzed. The results showed that *SLC25A5* is located on chromosome X, *ACSF2* on chromosome 17, *MFF* on chromosome 2, and *PMAIP1* on chromosome 18 (**Figure 4C**). Additionally, subcellular localization analysis provided insights into the potential roles of these biomarkers in PE pathogenesis. *ACSF2* and *SLC25A5* were localized to cellular membrane structures, suggesting their involvement in membrane-related processes associated with PE. In contrast, *MFF* and *PMAIP1* were localized to the cell nucleus, indicating their potential involvement in nuclear processes linked to the disease (**Figure 4D**).

### 3.5 Identification and characterization of PE biomarkers

**Figure 5:**
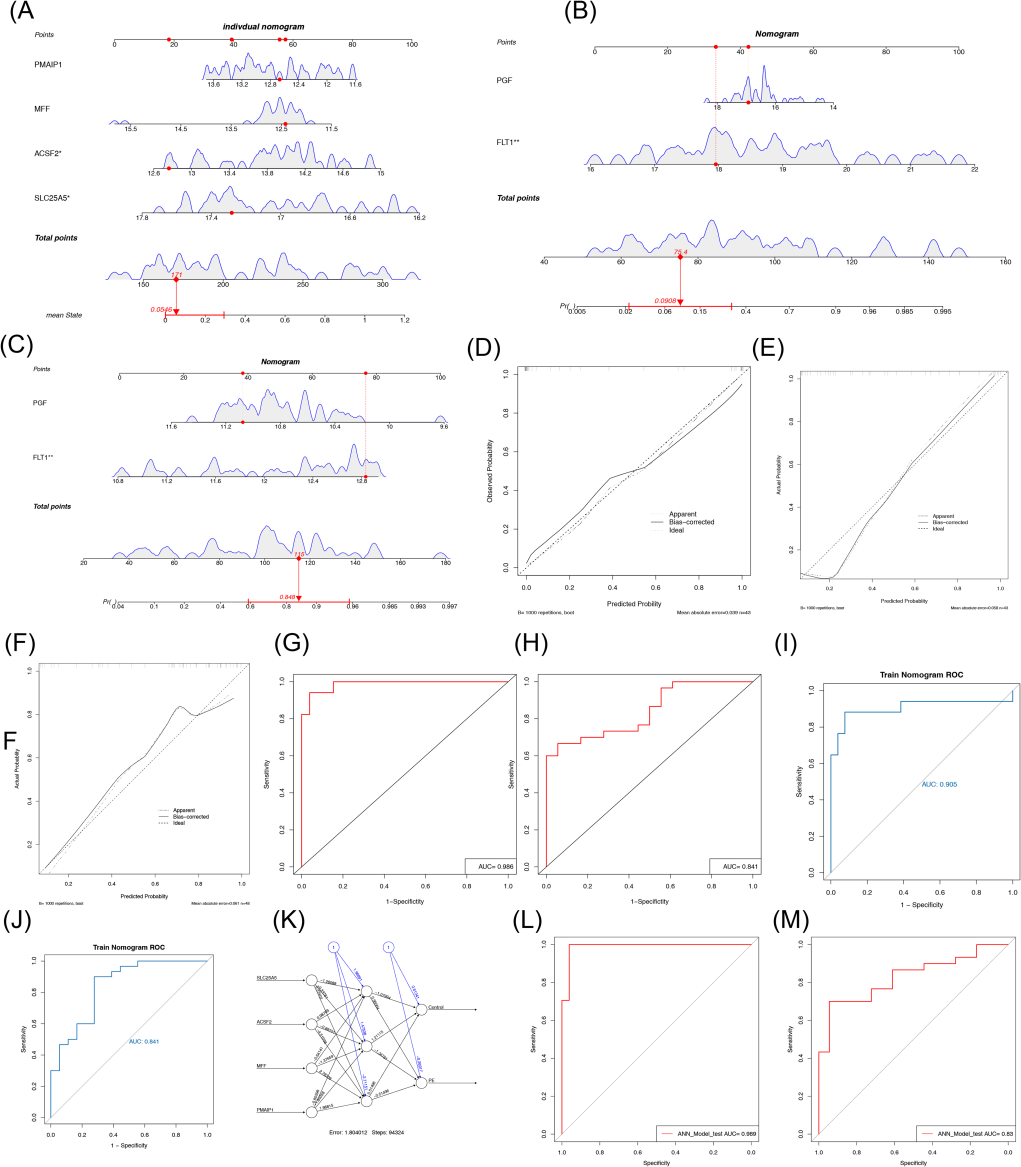
Evaluation and validation of the nomogram and artificial neural network diagnostic PE model. **(A)** Nomogram model of biomarkers. **(B)** Calibration curve of the model. **(C, D)** ROC curve of the model in the GSE10588 set and GSE98224 set. **(E)** Nomogram model of FLT1 and PGF in the GSE10588 dataset. **(F)** Calibration curves of FLT1 and PGF diagnostic models in the GSE10588 dataset. **(G)** ROC curves of FLT1 and PGF diagnostic models in the GSE10588 dataset. **(H)** Nomogram model of FLT1 and PGF in the GSE98224 dataset. **(I)** Calibration curves of FLT1 and PGF diagnostic models in the GSE98224 dataset. **(J)** ROC curves of FLT1 and PGF diagnostic models in the GSE98224 dataset. **(K)** Structural diagram of the artificial neural network composed of the five biomarkers. **(L, M)** ROC curve for artificial neural network evaluation in the GSE10588 set and GSE98224 set. PE, pre-eclampsia; ROC, receiver operating characteristic. *p < 0.05, **p < 0.01.

To further explore the relationship between each biomarker and the occurrence of PE, a nomogram was constructed on the basis of multiple logistic regression using the rms package in R. The nomogram assigned scores to each biomarker according to its expression level, and the total score was used to predict the probability of a PE diagnosis (**Figure 5A**). Calibration curve analysis indicated good model calibration, with an average error of 0.039 (**Figure 5D**). The area under the ROC curve (AUC) for the GSE10588 dataset was greater than 0.7, demonstrating the model’s strong discriminatory power (**Figures 5G, H**). The model robustness was further validated in the GSE98224 dataset, where the ROC curve also showed an AUC greater than 0.7 (**Figure 5I**). Nomograms were constructed separately for the two datasets (**Figures 5B, C**), and the calibration curves revealed mean errors of 0.058 and 0.0611, respectively (**Figures 5E, F**). In the GSE10588 dataset, the AUC value was 0.905 (0.783–1.000) (**Figure 5I**), and, in the GSE98224 dataset, the AUC value was 0.841 (0.720–0.961) (**Figure 5J**), indicating the accuracy of the nomogram models. The diagnostic potential of the biomarkers screened in this study was found to be superior to the results of FLT1 and PGF in the training set (AUC = 0.985 vs. AUC = 0.905).

Furthermore, an ANN diagnostic model was constructed on the basis of the biomarker data from the GSE10588 dataset. The weights of the four biomarkers in the ANN model ranged from −1.26 to 0.99, with *SLC25A5* having a weight of −1.26088, *ACSF2* having a weight of 0.98769, *MFF* having a weight of −0.64141, and *PMAIP1* having a weight of −0.85598 (**Figure 5K**). The ROC curve for the ANN model in the GSE10588 dataset yielded an AUC value of 0.989, indicating the model’s accuracy in distinguishing PE from normal samples (**Figure 5L**). In the GSE98224 dataset, the AUC value was 0.83, further confirming the robustness of the model in predicting PE (**Figure 5M**).

### 3.6 Impact of differential expression of PE biomarkers on pathways

**Figure 6:**
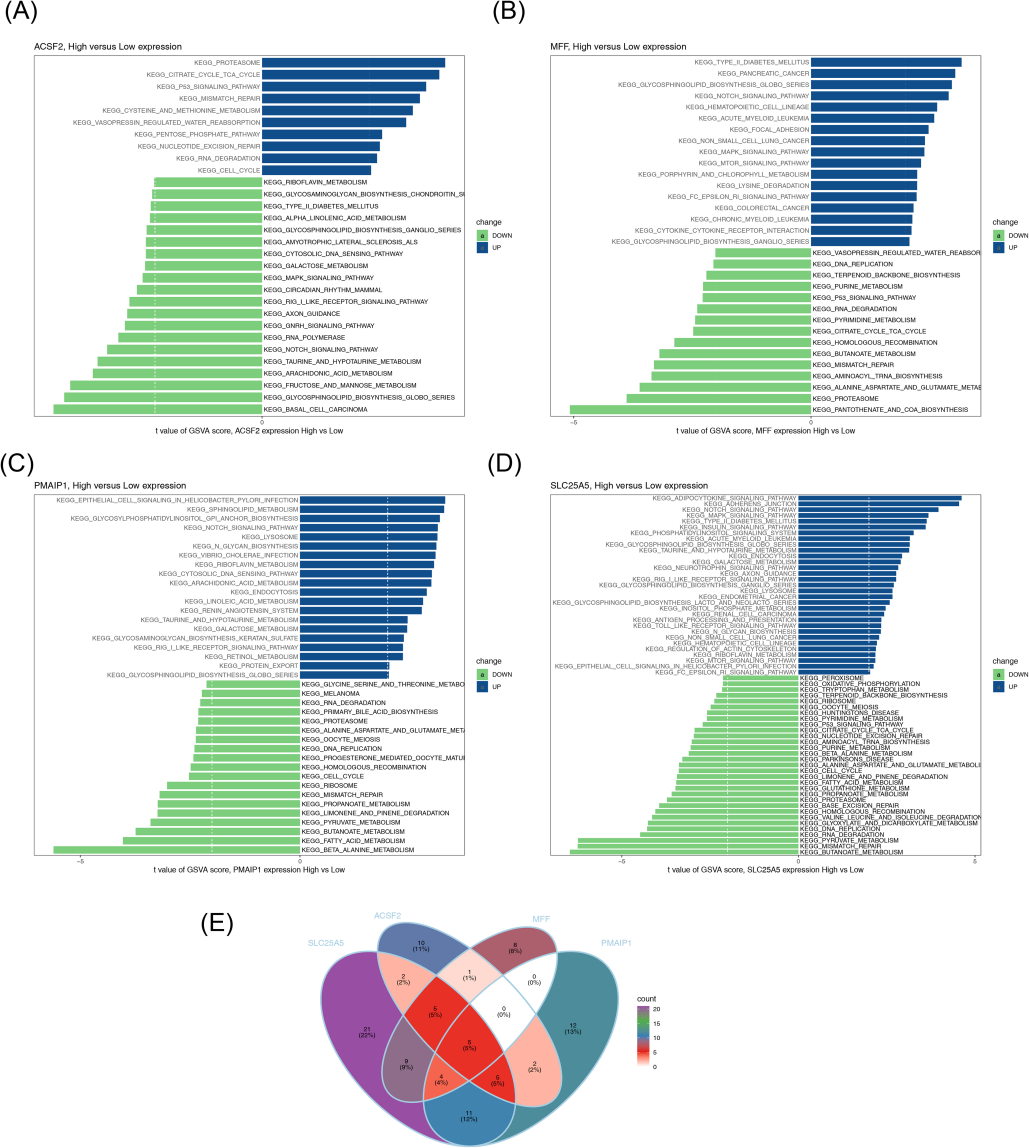
GSVA. **(A–D)** KEGG pathways with significant differences between high– and low–biomarker expression groups. Blue represents pathways activated in the high expression group, and green represents pathways activated in the low expression group. **(E)** Venn diagram of KEGG pathways shared by four groups. GSVA, gene set variation analysis; KEGG, Kyoto Encyclopedia of Genes and Genomes.

To further explore the impact of differential expression of biomarkers on KEGG pathways, the four biomarkers *(SLC25A5, ACSF2, MFF*, and *PMAIP1*) were categorized into high- and low-expression groups based on their median expression levels in the GSE10588 dataset. GSVA was conducted using the c2.cp.kegg.v2023.1.Hs.symbols.gmt gene set (containing 186 gene sets in total). The GSVA score for each KEGG pathway was calculated, and differential signaling pathways were screened using a threshold of |t| > 2 and p < 0.05 *via* the limma package. The analysis revealed five pathways with differential activation in both high- and low-expression groups of the biomarkers: mismatch repair, RNA degradation, Notch signaling pathway, proteasome, and glycosphingolipid biosynthesis globo series (**Figures 6A–E**; **Supplementary Figures S1**-**S5**).

### 3.7 Differential immune cells in PE samples and controls and their correlations

**Figure 7:**
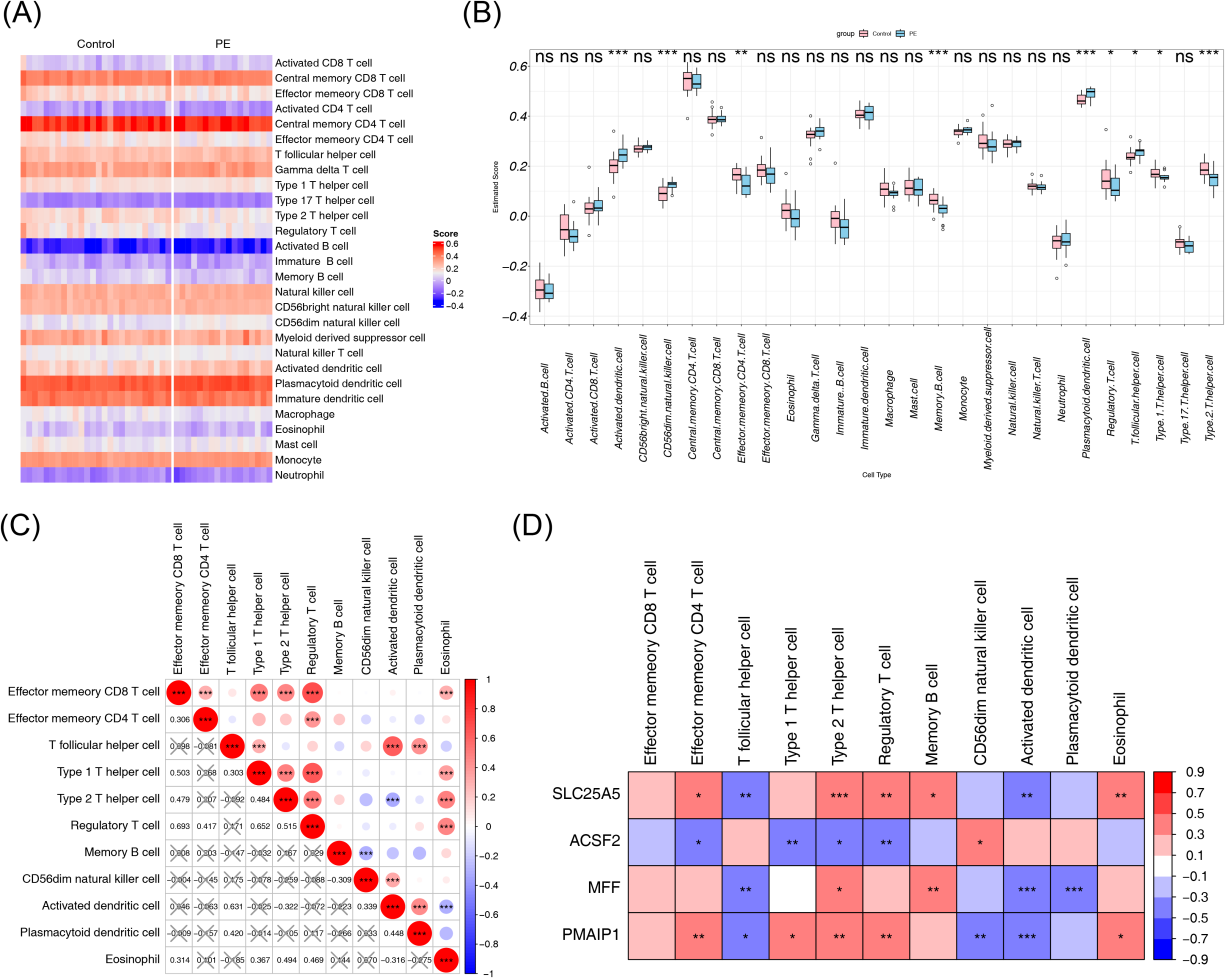
Immune infiltration analysis. **(A)** Heatmap of the immune cell composition score for each sample, with red indicating higher scores and blue indicating lower scores. **(B)** Differences in immune cell content between the PE and control groups. **(C)** Correlation heatmap of differential immune cells, with the correlation coefficient in the lower left corner, and X representing p > 0.05. **(D)** Correlation heatmap between biomarkers and differential immune cells. NS, non-significant, p > 0.05; *p < 0.05; **p < 0.01; ***p < 0.001. PE, pre-eclampsia.

To analyze immune differences between the PE and normal samples in the GSE10588 dataset, the composition scores of each immune cell type were computed and displayed in a heatmap (**Figure 7A**). Among the 28 immune cell types analyzed, 11 showed significant differences between the PE and normal groups. Activated dendritic cells, CD56dim natural killer cells, plasmacytoid dendritic cells, and T follicular helper cells had higher scores in the PE group, whereas the other differential immune cells showed the opposite trend (**Figure 7B**). Correlation analysis of the differential immune cells revealed that regulatory T cells and effector memory CD8 T cells had the strongest positive correlation (cor = 0.693), whereas activated dendritic cells showed the strongest negative correlation with type 2 T helper cells (cor = −0.322) (**Figure 7C**). To explore the relationship between biomarkers and differential immune cells, the correlation between the four biomarkers and the 11 differential immune cells was analyzed. Type 2 T helper cells showed significant correlations with all four biomarkers (|cor| > 0.3, p < 0.05), suggesting that these biomarkers may serve as potential targets for immunotherapy (**Figure 7D**).

### 3.8 Regulatory mechanisms and potential drugs for PE

**Figure 8:**
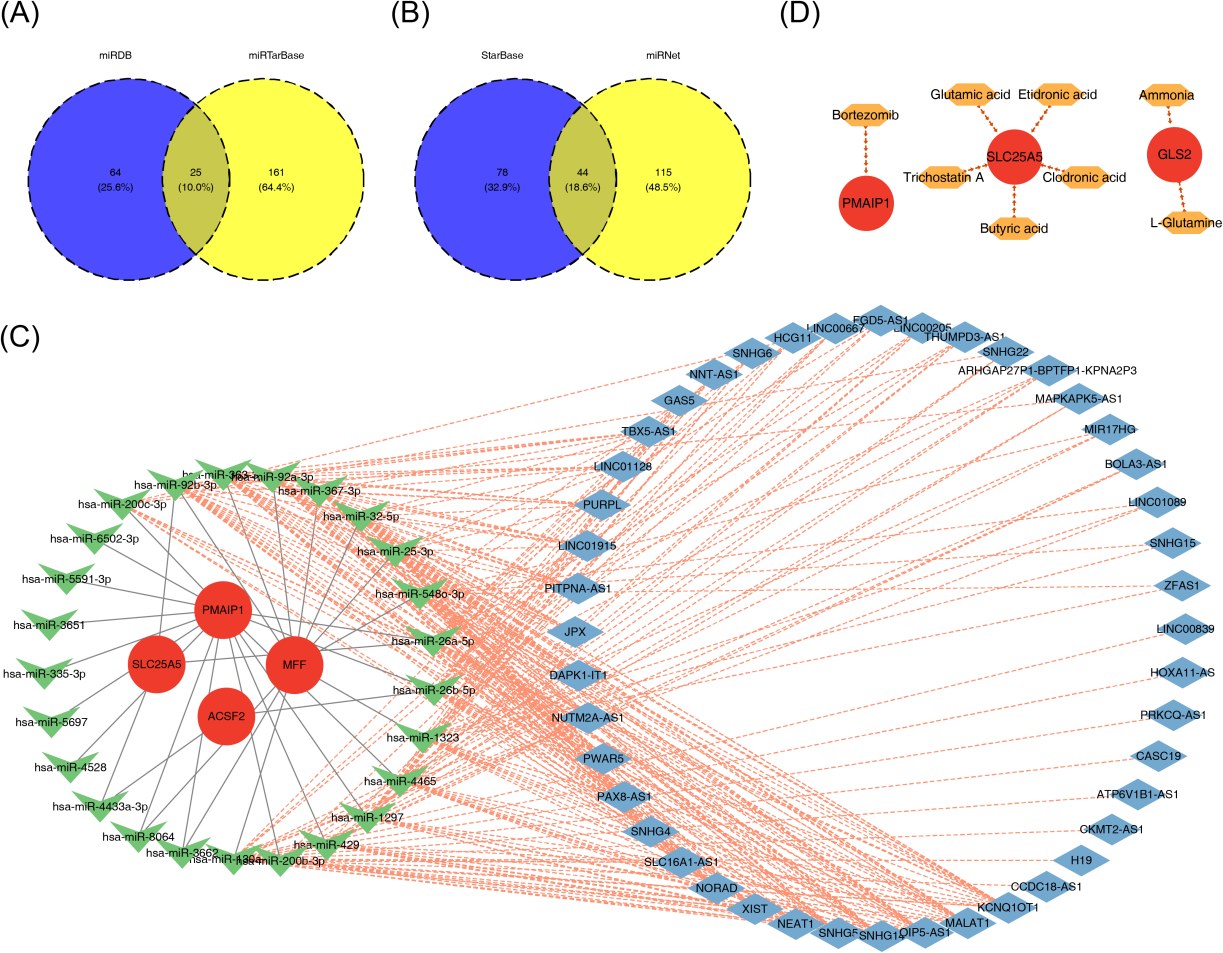
The ceRNA regulatory network and drug prediction. **(A, B)** Venn diagrams of shared miRNAs **(A)** and common lncRNAs **(B)** predicted by biomarkers. **(C)** The lncRNA–miRNA–mRNA regulatory network. **(D)** Drug–mRNA network, with red circles indicating biomarkers and orange hexagons indicating drugs. CeRNA, competing endogenous RNAs; miRNA, microRNA; lncRNA, long non-coding RNA.

To understand the regulatory mechanisms of the biomarkers, databases were utilized to predict corresponding regulatory factors. The miRDB and miRTarBase databases were used to predict miRNAs targeting the four biomarkers, resulting in 89 and 187 miRNAs, respectively. The intersection of these predictions yielded 25 miRNAs that could potentially regulate the biomarkers (**Figure 8A**). Using the StarBase and miRNet databases, 122 and 159 lncRNAs were predicted, respectively, based on the 25 miRNAs. The intersection of these lncRNAs resulted in a final set of 44 lncRNAs (**Figure 8B**). A lncRNA–miRNA–mRNA regulatory network was then constructed, with *KCNQ1* Opposite Strand/Antisense Transcript 1 (*KCNQ1OT1*) being identified as a regulator of *ACSF2* expression *via* modulation of hsa-miR-200b-3p (**Figure 8C**). Finally, drug predictions based on the biomarkers were conducted using relevant databases. Potential drug candidates for the treatment of PE included clodronic acid, etidronic acid, glutamic acid, L-glutamine, ammonia, bortezomib, trichostatin A, and butyric acid (**Figure 8D**).

### 3.9 Validation of the biological indicators of the screening

#### 3.9.1 Real-time quantitative polymerase chain reaction to verify the expression of biomarkers in PE

**Figure 9:**
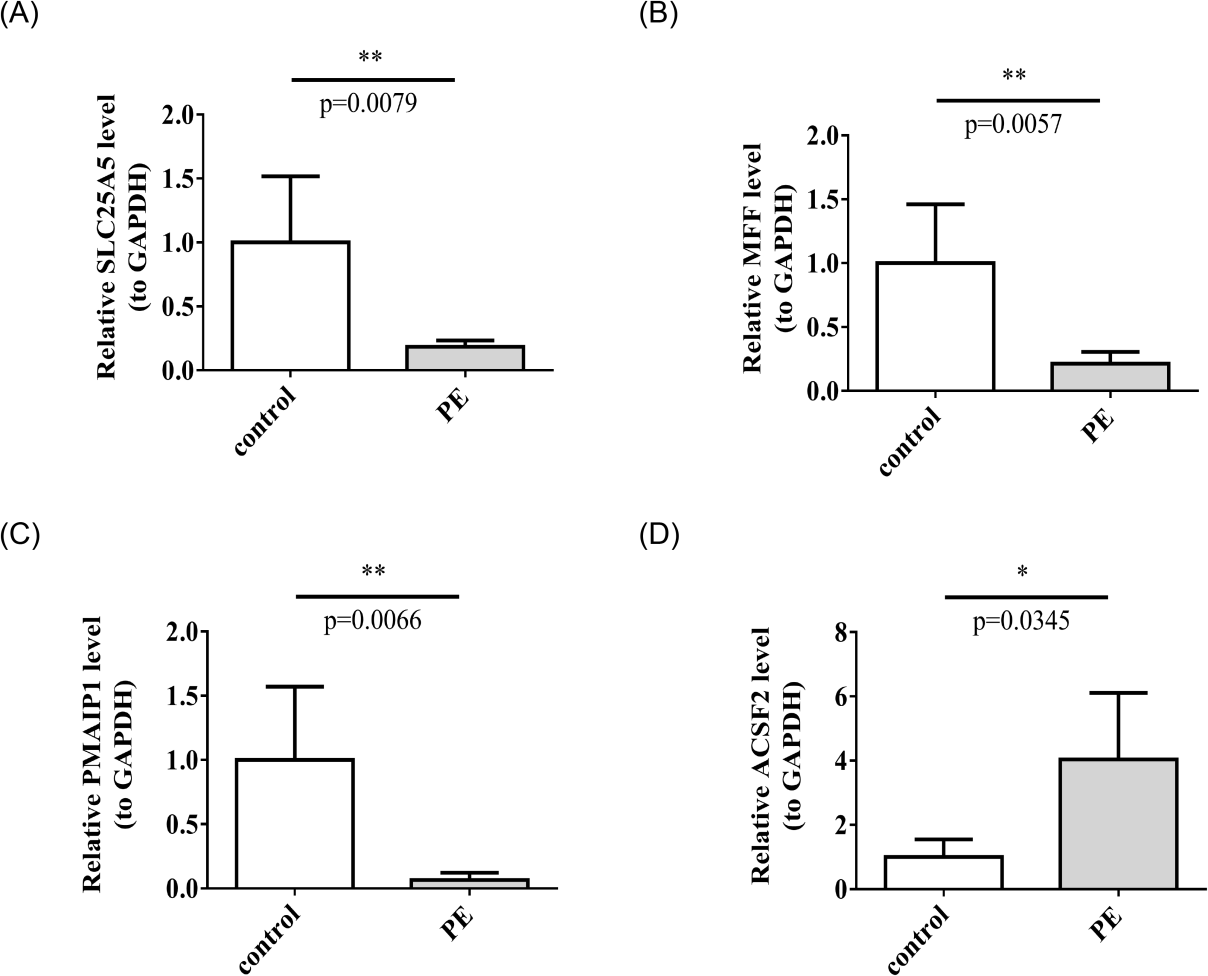
The mRNA expression levels of *SLC25A5* **(A)**, *MFF* **(B)**, *PMAIP1* **(C)**, *ACSF2* **(D)**. in control and PE samples by RT-qPCR. RT-qPCR, real-time quantitative polymerase chain reaction. *p < 0.05, **p < 0.01.

Gene expression patterns of *SLC25A5, MFF, PMAIP1*, and *ACSF2* in relation to PE were assessed using clinical RT-qPCR analysis. The results indicated that *SLC25A5, MFF*, and *PMAIP1* were downregulated in the PE group, whereas *ACSF2* was upregulated, reflecting distinct expression profiles for these genes in the context of PE (**Figures 9A–D**). These results aligned with the data obtained from the dataset.

#### 3.9.2 Immunofluorescence assay to verify the expression of biomarkers in the placenta of normal pregnancy and PE

**Figure 10:**
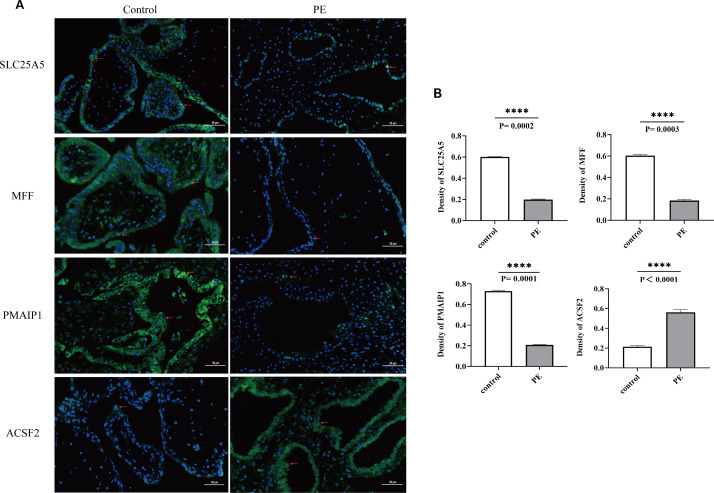
**(A)** Blue indicates the nucleus, green indicates the expressed target protein, and red arrows highlight the target protein expression sites in the cell. **(B)** Optical density analysis of immunofluorescence images for the four biomarkers. The AOD of SLC25A5, MFF, PMAIP1, and ACSF2 was significantly lower than in the control group, whereas ACSF2 showed significantly higher expression than in the control group. ****p < 0.0001; AOD, average optical density.

Immunofluorescence assays were conducted to examine the expression of *SLC25A5, MFF, PMAIP1*, and *ACSF2* in placentas from both PE and normal pregnancies. *SLC25A5* and *MFF* were localized to the cytoplasm and cell membrane of trophoblast cells, whereas *ACSF2* and *PMAIP1* were exclusively present in the cytoplasm. In the PE group, the expression intensity of *SLC25A5*, *MFF*, and *PMAIP1* was notably lower than in the control group, with significantly reduced average optical density values. Conversely, *ACSF2* exhibited enhanced expression and a significantly higher average optical density in the PE group, indicating distinct expression patterns for these genes in the context of PE (**Figures 10A, B**).

#### 3.9.3 Western blot to verify the expression of biomarkers in the placenta of normal pregnancy and PE

**Figure 11:**
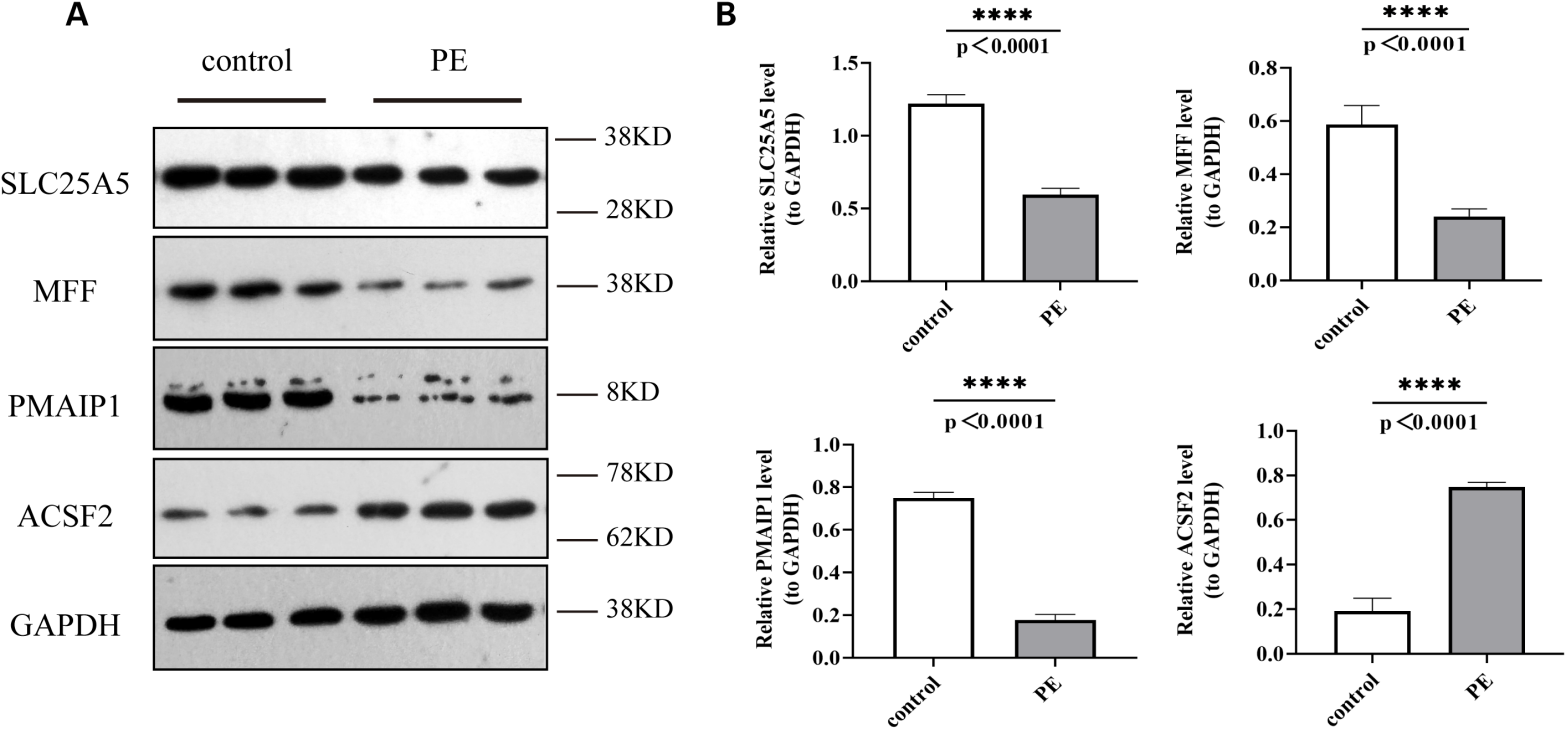
Protein expression levels of *SLC25A5, MFF, PMAIP1*, and *ACSF2* in control and PE samples by Western blot. **(A)** Western Blot (WB) results; **(B)** Quantitative analysis of WB results. WB, Western blot. ****p < 0.0001.

Western blot analysis confirmed these observations, revealing decreased expression levels of *SLC25A5, MFF*, and *PMAIP1* and an increased expression of *ACSF2* in the PE group compared to that in the control group (**Figures 11A, B**).

## 4 Discussion

PE, a pregnancy-specific complication, is a major cause of maternal and fetal morbidity and mortality, contributing to 4.6% of pregnancy-related complications (4). The pathogenesis of PE involves a range of factors, including placental hypoxia and ischemia, oxidative stress, inflammatory response, angiogenesis dysfunction, immune dysregulation, and their complex interactions (7). Among these, impaired trophoblast cell proliferation and abnormal invasion contribute to insufficient spiral artery remodeling, resulting in placental ischemia and hypoxia, which is considered a key pathogenic mechanism (45). Numerous studies have emphasized that PCD plays a critical role in trophoblast damage (46). Mitochondria, as central organelles in PCD, are involved in releasing or recruiting specific cell death promoters (47). Mitochondrial dysfunction, which disrupts intracellular energy metabolism, may trigger inflammation and vascular abnormalities, leading to trophoblast cell death (46). These insights open new avenues for exploring the pathogenesis of PE.

In this study, 1,438 DEGs were identified using bioinformatics, integrating public database data with clinical samples. After intersecting these genes with 1,136 MRGs and 1,548 PCD-related genes, 14 DE-mtPCD genes were obtained. GO and KEGG enrichment analyses of these DE-mtPCD genes revealed functional enrichment related to transmembrane transport, mitochondria, membrane permeability, peroxisome structures, apoptotic complexes, electron transfer, and redox processes. KEGG pathways associated with apoptosis, such as the p53 and apoptosis signaling pathways, were also identified. Previous research has indicated that p53-mediated trophoblast apoptosis is linked to the etiology of PE (48). The apoptosis pathway, which regulates PCD, is a terminal pathway for nearly all cell types.

To identify biomarkers involved in the onset of PE, this study applied three machine learning methods to select 10 key genes. Expression differences of these candidate biomarkers were verified in both the training and validation sets, ultimately identifying four genes—*SLC25A5, ACSF2, MFF*, and *PMAIP1*—as potential diagnostic biomarkers for PE. Specifically, *SLC25A5, MFF*, and *PMAIP1* were downregulated in the PE group, whereas *ACSF2* was upregulated across both datasets. *SLC25A5*, known as adenine nucleotide translocator 2, is critical for ATP/ADP exchange and plays a significant role in various diseases. High expression of *SLC25A5* is associated with poor prognosis in MRG studies (48). Furthermore, exposure to perfluorooctane sulfonate has been shown to impair trophoblast migration, invasion, and vascular formation, reducing *SLC25A5* expression in both the placenta and JEG-3 cells. Animal and *in vitro* experiments confirmed that mitochondrial dysfunction mediated by *SLC25A5* in trophoblast cells induces these pathophysiological effects, ultimately leading to PE (49). These observations suggest that *SLC25A5* may contribute to placental dysfunction by affecting mitochondrial function, warranting further investigation.

*ACSF2*, a member of the acyl-CoA synthetase (*ACS*) family, catalyzes the sulfur esterification of acyl thioesters to form coenzyme A, which plays a central role in cellular lipid metabolism (50). Mitophagy, the selective degradation of damaged mitochondria, is essential for maintaining mitochondrial homeostasis and generating ATP to support various cellular functions (51). Inhibition of *ACSF2* in Human renal cortex proximal convoluted tubule epithelial cells-2 (HK-2) cells has been shown to reduce cellular lipid peroxidation, enhance mitophagy, restore mitochondrial function, and protect against ischemia-reperfusion–induced acute kidney injury (52). In another study, overexpression of *DRAM1* in mice enhanced mitophagy, improved placental mitochondrial function in PE mice, and significantly reduced blood lipid and urinary protein levels (53). These observations suggest that *ACSF2* may alleviate PE symptoms by enhancing mitophagy and improving mitochondrial function, positioning it as a potential therapeutic target for PE. Additionally, *ACSF2* is significantly associated with immune-related pathways such as Toll-like receptor signaling, Nuclear Factor κ-Light-Chain Enhancer of Activated B Cells (NF-κB) signaling, and Nucleotide binding oligomerization domain (NOD)-like receptor signaling (54–56), all of which are implicated in the pathogenesis of PE, further supporting the potential involvement of *ACSF2* in PE onset (57–59). Future experiments are needed to clarify the specific role of *ACSF2* in PE pathogenesis.

*MFF*, located on the outer mitochondrial membrane, is crucial for activating mitochondrial fission and mediating mitochondrial death. Studies have shown that genetic deletion of *MFF* suppresses pro-inflammatory responses, renal tubular oxidative stress, and renal cell death, significantly mitigating renal failure caused by ischemic acute kidney injury (AKI) (60). *MFF* mediates mitochondrial fission by facilitating the translocation of dynein-related protein 1 (*Drp1*) from the cytosol to mitochondria and negatively regulates calcium (Ca^2+^) transport from the ER to mitochondria. *MFF* deficiency leads to mitochondrial Ca^2+^ overload, which triggers excessive ROS production, impedes mitochondrial biogenesis, and results in encephalopathy (61).

*PMAIP1*, a member of the pro-apoptotic *BCL-2* family (specifically the BH3 subfamily), regulates apoptosis and proliferation in various tumor cells (62–64). *PMAIP1* contains a binding site for p53, which directly interacts with this site to promote *PMAIP1* transcription and protein expression, mediating apoptosis (65). Although *PMAIP1* is primarily recognized as a mediator of p53-induced apoptosis, it has been found that hypoxia-inducible factor 1α can bind to the hypoxia response element upstream of the PMAIP1 promoter, thereby activating *PMAIP1* transcription and confirming its role in apoptosis through a p53-independent pathway (66).

Although *SLC25A5, ACSF2, MFF*, and *PMAIP1* have not been previously linked to PE, they are known to significantly influence mitochondrial function and tumor cell death. Given the similarities between trophoblast cell invasion and proliferation and tumor cell behavior, it is plausible that these biomarkers may play a role in mediating trophoblast cell death during PE pathogenesis.

Building on the previous results, the gene expression of *SLC25A5, MFF, PMAIP1*, and *ACSF2* was further assessed in placental tissues from both PE and normal pregnancies using RT-qPCR. The findings revealed a reduction in the expression levels of *SLC25A5, MFF*, and *PMAIP1*, whereas *ACSF2* expression was elevated in the PE group. These clinical validation results were consistent with the dataset analysis, suggesting that *SLC25A5, MFF, PMAIP1*, and *ACSF2* could serve as novel potential targets for the prevention and treatment of PE.

Further analyses using a nomogram and ANN demonstrated the strong discriminatory ability of these biomarkers in distinguishing between PE and normal groups, underscoring their potential for effective PE diagnosis. In medical diagnostics, the AUC is a critical metric for evaluating test accuracy, with AUC values approaching 1 indicating greater diagnostic reliability (67). In this study, the AUC for the biomarkers *SLC25A5, MFF, PMAIP1*, and *ACSF2* was 0.986, highlighting their exceptional accuracy in predicting PE. While traditional biomarkers have proven useful in PE diagnosis, the present findings suggest that these novel biomarkers may offer superior diagnostic performance. Clinically, a higher AUC provides substantial benefits by more accurately distinguishing true patients with PE. This approach reduces the misclassification of healthy individuals as patients with PE (false positives) and minimizes missed diagnoses (false negatives), offering a more reliable foundation for early diagnosis, timely intervention, and improved patient outcomes.

Further GSVA identified five pathways that exhibited significant differences between high- and low-expression groups of the four biomarkers, including mismatch repair, RNA degradation, Notch signaling pathway, proteasome, and glycosphingolipid biosynthesis (Globo and Isoglobo Series). Mismatch repair, a critical DNA repair pathway, is involved in mitosis, meiosis, cell apoptosis, immunoglobulin gene rearrangement, and somatic hypermutation. Disruption of this pathway may be central to the PCD observed in PE cells (68). In RNA regulation, RNA degradation, particularly of polyadenylated RNA, occurs rapidly during early apoptosis, potentially serving as a marker of cell death and being associated with mitochondrial release proteins (69). The Notch signaling pathway plays a pivotal role in determining cell fate and regulating cell differentiation, proliferation, and apoptosis through interactions between Notch ligands and receptors (70). This pathway is essential for normal placental and trophoblast development, promoting successful pregnancy (71). Notch1, in particular, is critical for the proliferation and survival of extravillous trophoblast precursors, and defects in trophoblast differentiation are linked to severe pregnancy complications, including PE (72). The proteasome pathway, involved in protein modification and degradation, is also implicated in regulating apoptosis through various signaling pathways, such as the ubiquitin-proteasome and autophagy pathways (73). These pathways are interconnected with apoptosis and have been linked to PE pathogenesis. Therefore, these four biomarkers may contribute to mediating trophoblast apoptosis and the development of PE, although further exploration of the underlying mechanisms is warranted.

Gene regulatory networks are essential for the regulation of gene expression and play a significant role in disease development. lncRNAs and Circular RNAs (circRNAs) can modulate miRNA activity, influencing downstream mRNA expression and impacting conditions such as PE (74). In this study, a database was used to predict corresponding regulatory factors, resulting in the identification of 44 lncRNA–miRNA–mRNA interaction networks, which were visualized. Although no prior studies have documented these specific regulatory networks in PE, they represent an area of considerable potential for further investigation into the regulatory mechanisms underlying PE. Additionally, drug prediction based on these four biomarkers identified potential therapeutic candidates for PE treatment, including glutamic acid and clodronate. These findings offer promising targets for the development of therapeutic strategies for PE.

Extensive research has highlighted the involvement of immune imbalance in the pathophysiology of PE (75, 76). Several bioinformatics analyses have also indicated significant immune infiltration differences between PE and normal controls (77). In this study, immune variations between PE and normal samples were analyzed using the GSE10588 dataset, revealing that 11 immune cell types were significantly different between the two groups. The correlation analysis between these differential immune cells and the four biomarkers demonstrated that type 2 T helper (Th2) cells were significantly associated with all four biomarkers. Specifically, Th2 cells were positively correlated with *SLC25A5, MFF*, and *PMAIP1* and negatively with *ACSF2*. MFF interacts with *Drp1* to initiate mitochondrial division, which may influence the mitochondrial function and survival of Th2 cells (78). As a pro-apoptotic protein, *PMAIP1* (also known as *NOXA*) regulates the survival and function of memory CD4(+) Th1/Th2 cells by binding to anti-apoptotic proteins such as Mcl-1 and *Bcl2A1* (79). *SLC25A5* plays a role in apoptosis regulation by modulating mitochondrial membrane permeability, which could further impact Th2 cell survival (80). Moreover, *ACSF2* may influence the energy metabolism and overall function of Th2 cells by regulating lipid metabolism (52). These findings suggest that these four biomarkers modulate Th2 cell survival, function, and energy metabolism *via* distinct mechanisms, highlighting the potential role of Th2 cells in regulating the immune response in PE. After placental implantation in normal pregnancy, the early inflammatory Th1 immune response rapidly shifts to a Th2 anti-inflammatory response. Dominant Th2 immunity overcomes Th1 immunity at the placental implantation site, balancing Th1 activity to protect the fetus and support fetal and placental development. However, an enhanced Th2 response during pregnancy can contribute to or exacerbate autoimmune diseases (81). Other studies (82) have reported that, while Th2 cells increase in normal pregnancy circulation, they decrease in pre-eclamptic pregnancies. This dysregulation, often observed in the first month of PE, is accompanied by a rise in circulating and placental CD4^+^ Th1 cells, elevated pro-inflammatory cytokine levels, increased autoantibody production, and oxidative stress, suggesting that Th2 cells and related pathways may serve as potential targets for immunotherapy.

This study is the first to investigate the association between PE and mtPCD based on public databases. Through bioinformatics analysis, biomarkers of diagnostic value were identified, and related pathway analyses were conducted. However, several limitations must be acknowledged. Firstly, conclusions derived from bioinformatics analysis may be susceptible to bias. Bioinformatics heavily depends on existing databases and algorithms, and factors such as the accuracy, completeness of data sources, and the applicability of algorithms can influence the results. Thus, further clinical validation of the findings is essential. Although verification experiments, including PCR, immunofluorescence, and Western blot, were performed, the experimental validation remains incomplete. Additional experiments, such as Cell Mito stress Seahorse assays and blood analyses, were not conducted. Moreover, the small sample size used for experimental validation limits the representativeness and generalizability of the results. Furthermore, although potential drugs such as clodronic acid, etidronic acid, and glutamic acid were predicted, their effectiveness in PE samples was not evaluated. Finally, although the differential expression of biomarkers has been preliminarily validated, their biological roles in the pathogenesis of PE and their ability to predict disease severity or complications have not been comprehensively explored. This gap in understanding limits the potential for developing effective biomarker-based therapeutic strategies.

To address these limitations, future work will focus on further bioinformatics analyses and clinical validation of targeted experiments. By increasing the sample size and including samples from different races, regions, and lifestyle factors, this study aims to enhance the representativeness and generalizability of the study results. Additionally, experimental validation using blood samples will be incorporated to provide a more comprehensive exploration of the biomarkers’ characteristics and effects. Further, the correlation between biomarkers and genes highly expressed in hypertension will be analyzed to assess whether differential expression of biomarkers is influenced by hypertension. Extending the analysis of immune cell populations, particularly Th2 cells, could explore their role in PE. Advanced biotechnologies such as gene editing, cell function assays, and animal model construction could uncover the biological functions of these biomarkers in PE pathogenesis and the efficacy of potential drugs. This approach will provide a comprehensive molecular, cellular, and systemic analysis of the relationship between biomarkers and PE pathogenesis. Moreover, more cases of PE with varying severity and complications will be collected, further evaluating the predictive efficacy of these biomarkers for disease severity and complications by combining expression level data with clinical outcomes.

## 5 Conclusions

This study is the first to establish a link between mtPCD-related genes and PE. Four biomarkers—*SLC25A5, ACSF2, MFF*, and *PMAIP1*—associated with mtPCD were identified, demonstrating strong diagnostic potential for PE. Furthermore, the study has conducted preliminary investigations into the functional enrichment pathways, lncRNA–miRNA–mRNA regulatory network, immune infiltration, and drug predictions related to these biomarkers, revealing their substantial application potential. These biomarkers may not only serve as novel therapeutic targets, with the development of specific drugs or treatments potentially transforming disease outcomes, but also offer valuable tools for screening and assessing drug efficacy. These findings open new avenues for advancing the diagnosis and treatment of PE.

## Data Availability

The datasets presented in this study can be found in online repositories. The names of the repository/repositories and accession number(s) can be found in the article/**Supplementary Material**.

## Supplementary material

Supplementary Figure 1 Mismatch repair pathway in DNA repair processes.

Supplementary Figure 2 Glycosphingolipid biosynthesis in the globo series pathway.

Supplementary Figure 3 The Notch signaling pathway, a key determinant of cell fate.

Supplementary Figure 4 The proteasome pathway regulates cell apoptosis.

Supplementary Figure 5 RNA degradation pathway modulates RNA expression.

Supplementary Table 1 List of KEGG enrichment results.

Supplementary Table 2 List of LASSO regression coefficients for the 10 hub genes.

Supplementary Table 3 Chromosomal localization of the four biomarkers.

Supplementary Table 4 List of regression coefficients.

Supplementary Table 5 List of targeted drugs for the biomarkers.
