# Supplementary figures and images for: Identification and preliminary validation of biomarkers associated with mitochondrial and programmed cell death in pre-eclampsia
Source: Front Immunol. 2025 Jan 23;15:1453633. doi: 10.3389/fimmu.2024.1453633 (PMC11798957; doi:10.3389/fimmu.2024.1453633)

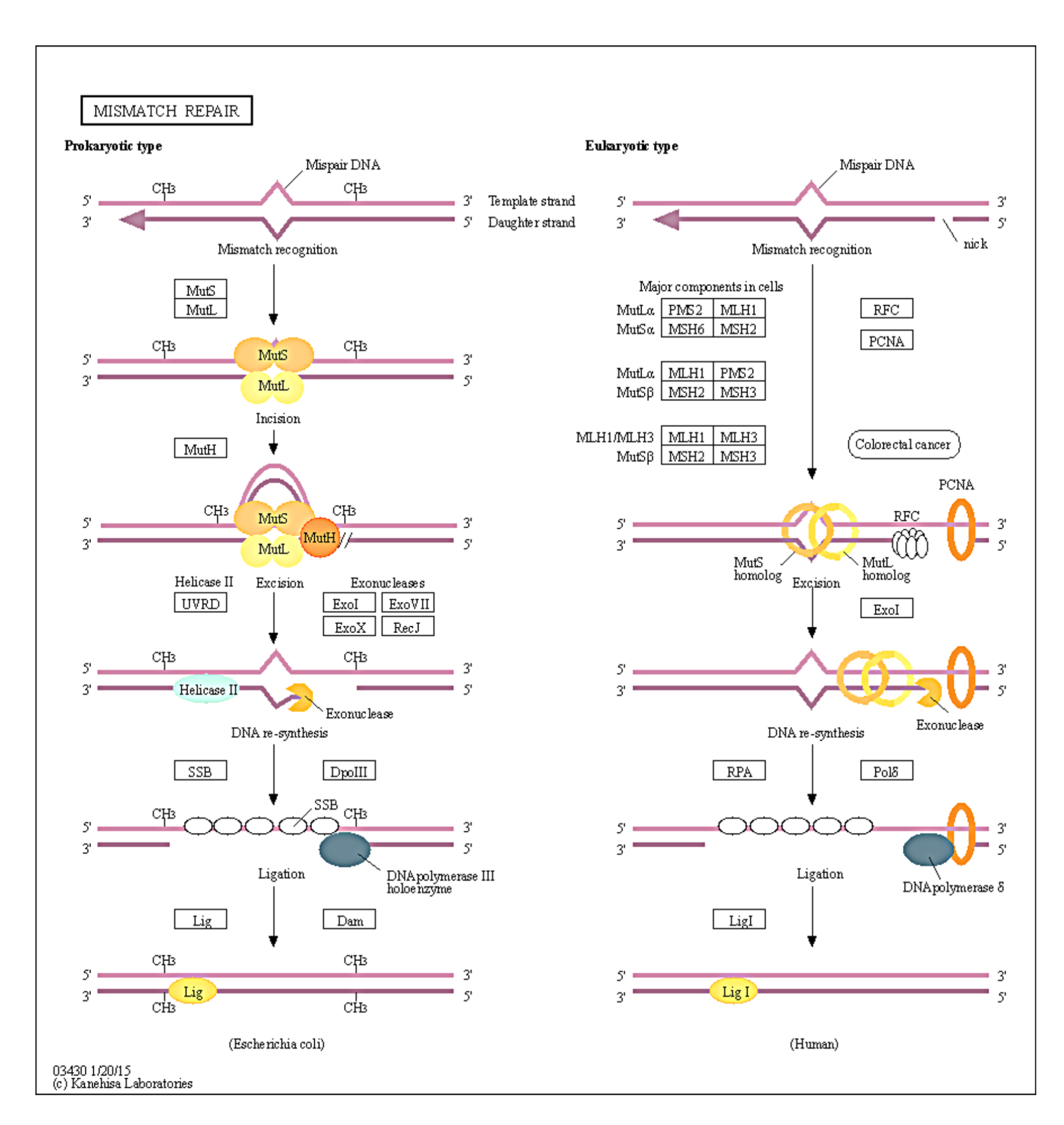

Supplement: Supplementary Figure 1 — Mismatch repair pathway in DNA repair processes. [file Image1.tif]

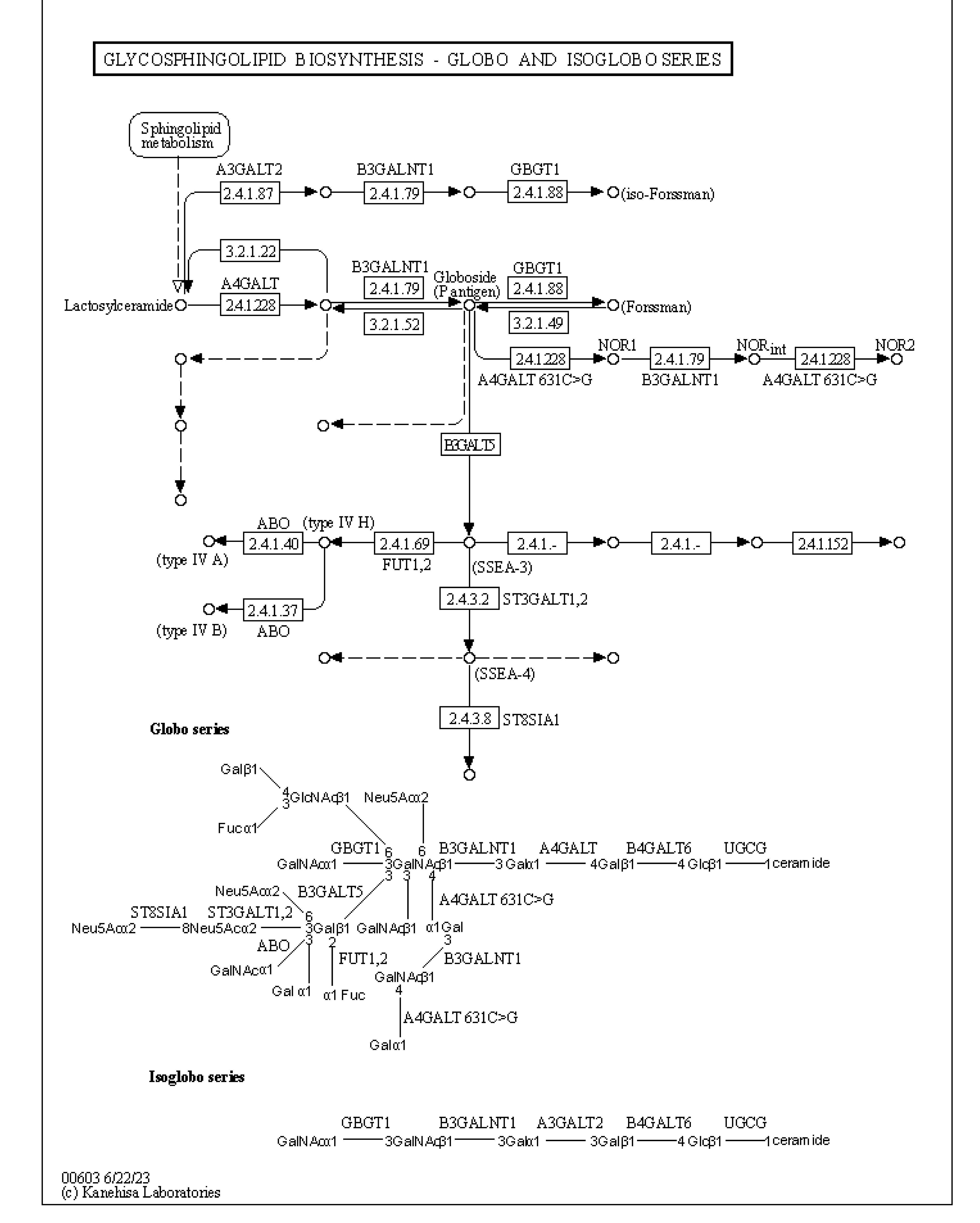

Supplement: Supplementary Figure 2 — Glycosphingolipid biosynthesis in the globo series pathway. [file Image2.tif]

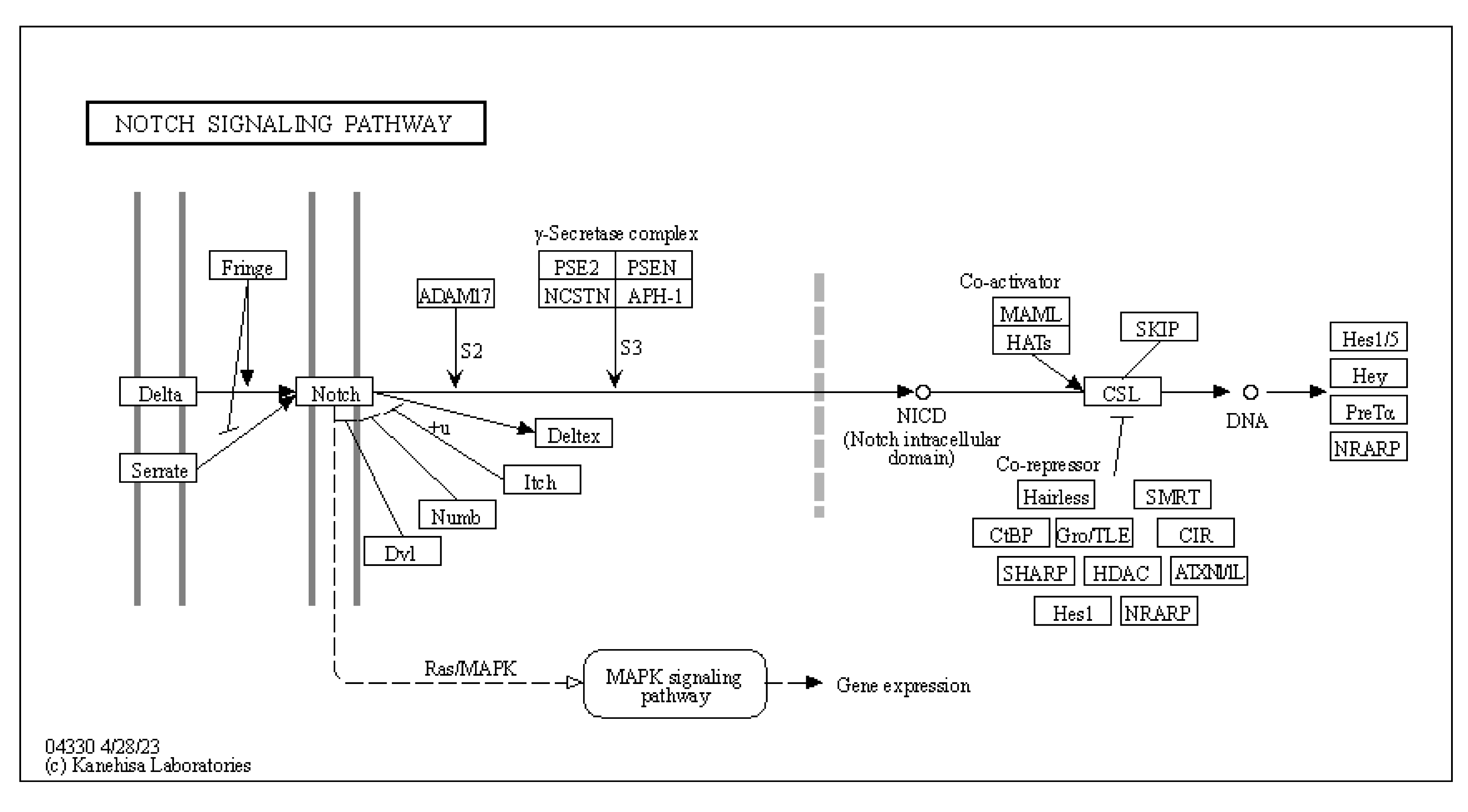

Supplement: Supplementary Figure 3 — The Notch signaling pathway, a key determinant of cell fate. [file Image3.tif]

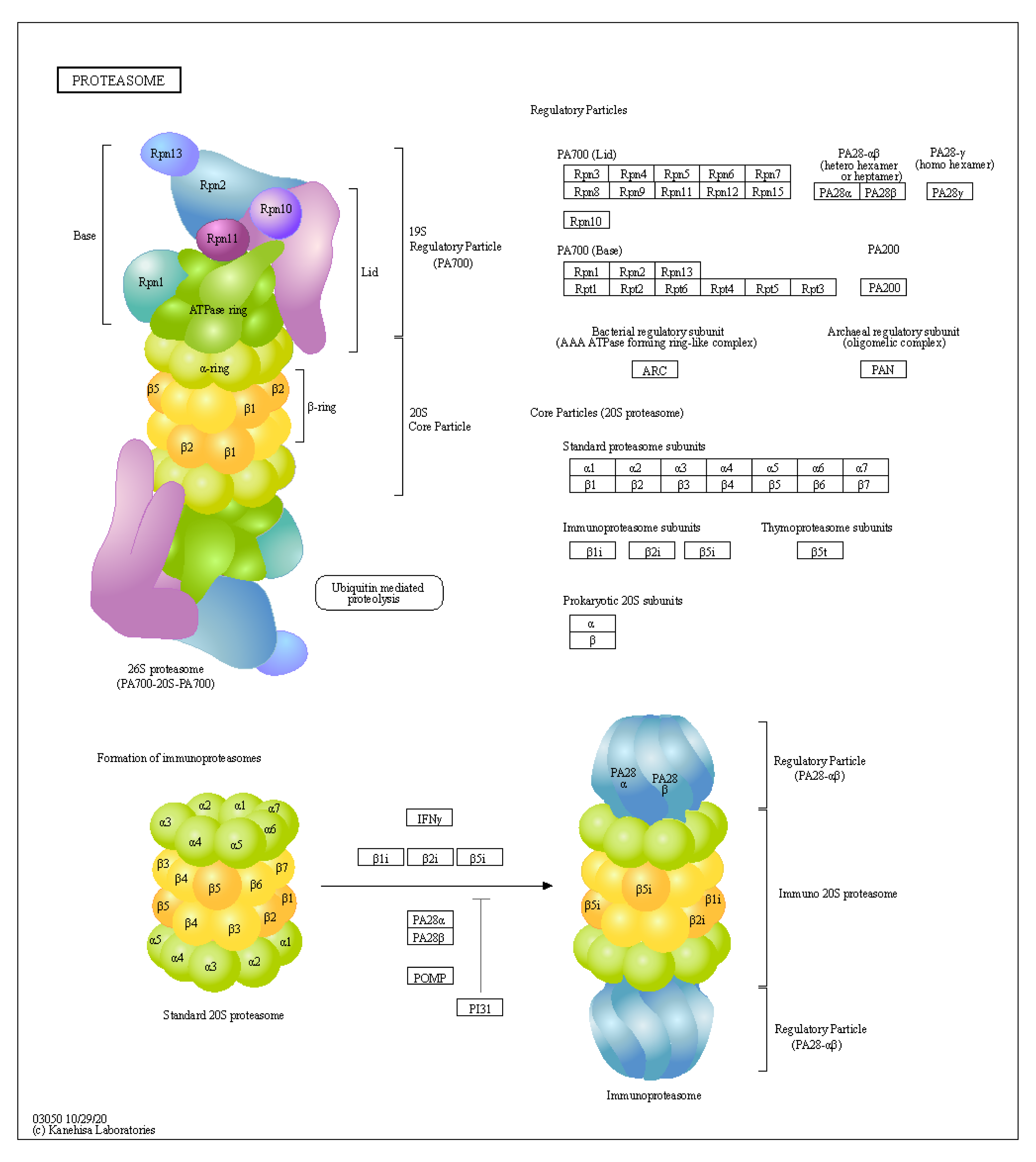

Supplement: Supplementary Figure 4 — The proteasome pathway regulates cell apoptosis. [file Image4.tif]

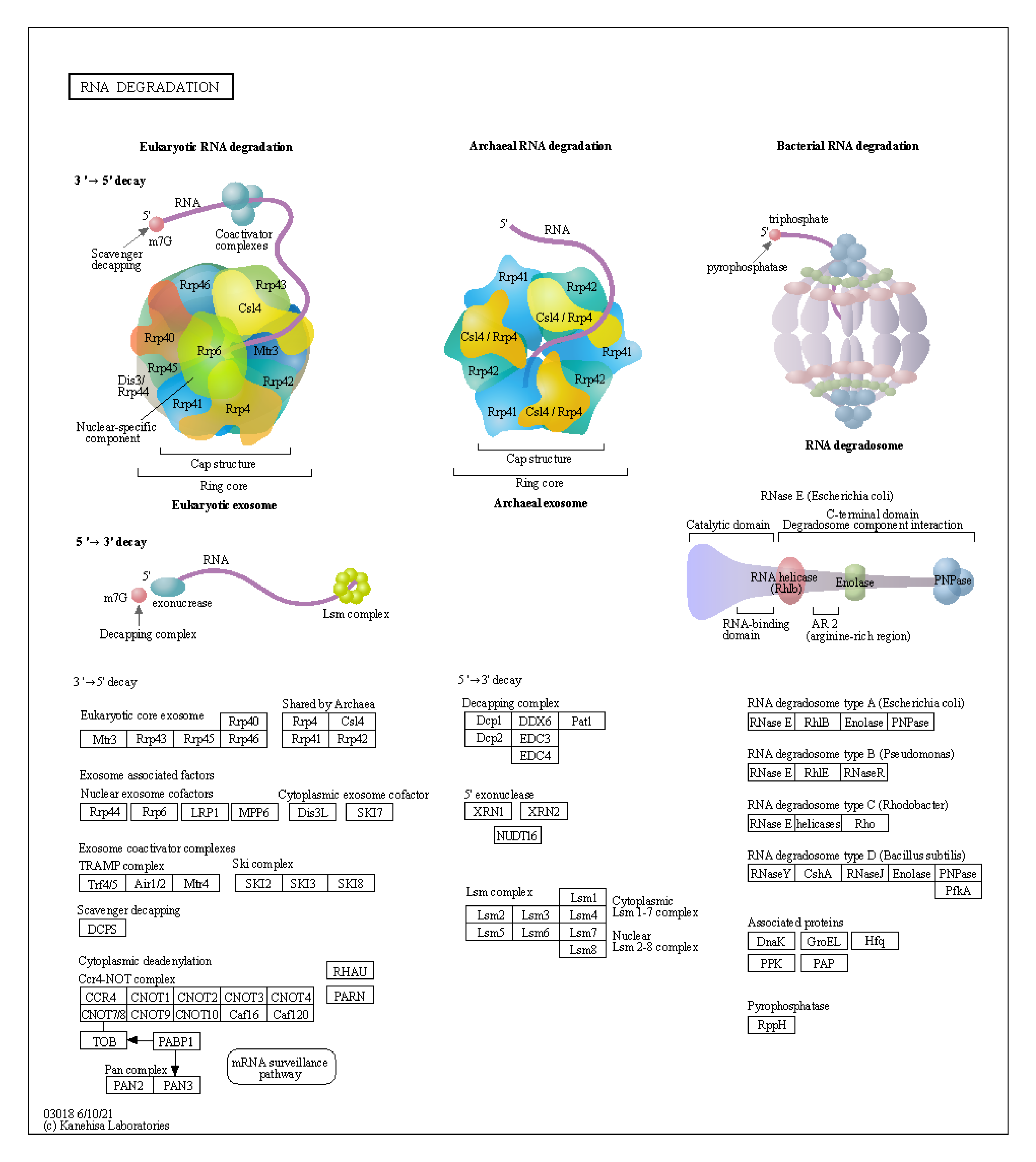

Supplement: Supplementary Figure 5 — RNA degradation pathway modulates RNA expression. [file Image5.tif]
